# Tuning aminopolycarboxylate chelators for efficient complexation of trivalent actinides

**DOI:** 10.1038/s41598-023-44106-6

**Published:** 2023-10-19

**Authors:** Corey D. Pilgrim, Travis S. Grimes, Clayn Smith, Colt R. Heathman, Jopaul Mathew, Santa Jansone-Popova, Santanu Roy, Debmalya Ray, Vyacheslav S. Bryantsev, Peter R. Zalupski

**Affiliations:** 1 Aqueous Separations and Radiochemistry, Idaho National Laboratory, Idaho Falls, ID 83415 USA; 2 Glenn T. Seaborg Institute, Idaho National Laboratory, Idaho Falls, ID 83415 USA; 3 Chemical Sciences Division, Oak Ridge National Laboratory, Oak Ridge, TN 37831 USA

**Keywords:** Nuclear chemistry, Coordination chemistry, Chemical synthesis, Inorganic chemistry

## Abstract

The complexation of trivalent lanthanides and minor actinides (Am^3+^, Cm^3+^, and Cf^3+^) by the acyclic aminopolycarboxylate chelators 6,6′-((ethane-1,2-diylbis–((carboxymethyl)azanediyl))bis–(methylene))dipicolinic acid (H_4_octapa) and 6,6'-((((4-(1-(2-(2-(2-hydroxyethoxy)ethoxy)ethyl)-1H-1,2,3-triazol-4-yl)pyridine-2,6-diyl)bis–(methylene))bis–((carboxymethyl)azanediyl))bis–(methylene)) dipicolinic acid (H_4_pypa-peg) were studied using potentiometry, spectroscopy, competitive complexation liquid–liquid extraction, and ab initio molecular dynamics simulations. Two studied reagents are strong multidentate chelators, well-suited for applications seeking radiometal coordination for *in-vivo* delivery and f-element isolation. The previously reported H_4_octapa forms a compact coordination packet, while H_4_pypa-peg is less sterically constrained due to the presence of central pyridine ring. The solubility of H_4_octapa is limited in a non-complexing high ionic strength perchlorate media. However, the introduction of a polyethylene glycol group in H_4_pypa-peg increased the solubility without influencing its ability to complex the lanthanides and minor actinides in solution.

## Introduction

At the end of its service in a commercial nuclear reactor the irradiated nuclear fuel consists of 95% uranium dioxide, 1% plutonium dioxide, roughly 3.5% fission products, and ≈ 0.5% minor actinides (An; predominantly Np, Am, and Cm)^1^. The majority of countries with established nuclear energy technologies manage used nuclear fuel as waste for a variety of socio-economic reasons. Globally, as our society continues to mature, the ethics of sustainability and environmental responsibility will continue to evolve, strengthening the argument for a more resourceful approach. In a closed nuclear fuel cycle option the actinides are reused in reactors to generate electric power^2^. This approach enables increased utilization of uranium resources, and helps manage the long-term fate of radioactive byproducts through a reduction of bulk volume, radiotoxicity, and decay heat load inside a geologic repository^3^.

Actinide recovery/recycle/isolation techniques center on the development of efficient sequestering reagents, capable of selective coordination of the 5*f* elements in aqueous effluents containing nearly a third of the periodic table^4–6^. A variety of metal ion complexants have been explored throughout the years, mainly targeting the rich redox chemistry of major actinides (U, Pu, and Np), and electronic properties of trivalent minor actinides (Am and Cm)^7^. The isolation of the 5*f* metals commonly employs solvent extraction methods, exploiting the chemical binding preferences to partition actinides into an immiscible liquid phase, and separate them away from unwanted metal ions. Efficient differentiation of trivalent minor actinides and trivalent lanthanides (Ln) is of particular importance as the 4*f* fission products are neutron poisons^1^, but selective binding of the trivalent An is obstructed by similar solution chemistry of the trivalent Ln^8^. The *f*-elements are collectively hard Lewis acids, highly hydrated spherical cations in solutions with compact *f*-orbitals, embedded in the interior of an ion. Similar ionic radii, contracting across the 4*f* and 5*f* series, yield trivalent cations of nearly equivalent charge density (e.g., Am^3+^ and Pm^3+^). Accordingly, such ions are indistinguishable to hard Lewis bases (oxygen donor ligands). The selective binding of trivalent actinides is realized due to the enhanced spatial extension of the 5*f* orbitals, relative to 4*f* orbitals, inviting stronger interaction with reagents containing less electronegative atoms (e.g., N and S)^8^.

**Figure 1:**
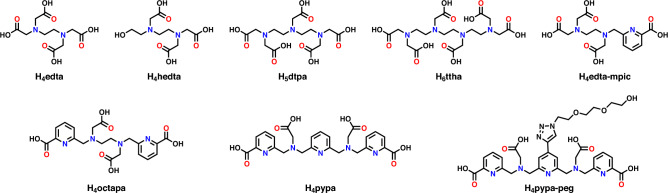
Chemical structures of different aminopolycarboxylate ligands.

The European strategy for trivalent An/Ln differentiation employs heterocyclic nitrogen donor ligands^9,10^. The two classes of such reagents, bis–triazinylpyridines, bis–triazinyl bipyridines, and bis–triazinyl phenanthrolines contain highly acidic amine binding sites, sustaining efficient An/Ln selectivity in mixtures containing molar quantities of nitric acid^9,10^. Recently, a bis–triazolylpyridine chelator was identified as a suitable choice, accomplishing the An^3+^/Ln^3+^ task in moderately acidic aqueous mixtures (0.25 M nitric acid)^11^. In the United States, acyclic aminopolycarboxylate *N*-donor ligands are utilized to facilitate An/Ln separations^2,12–14^. As with the heterocyclic nitrogen donors the presence of amine moieties enhances the stability of actinide complexes. Efficient group differentiation is accomplished if a multidentate reagent like diethylenetriamine-*N,N,N',N'',N''*-pentaacetic acid (H_5_dtpa) is employed^12,13^. The structure of H_5_dtpa is shown in Fig. 1. The weaker [Ln(dtpa)]^2−^ complexes dissociate, and the Lns are extracted into the non-aqueous environment. The stronger [An(dtpa)]^2−^ complexes remain in the aqueous layer. Although simple and eloquent in its original recipe, the aminopolycarboxylate (APC) approach offers a solution in buffered, mildly acidic (pH > 2) aqueous environment, where metal complexation by APCs is strong. The liquid–liquid distribution is also slow to attain equilibrium as low proton content inhibits the hydrogen ion catalyzed mechanism of metal complex dissociation^12,13^.

The operational pH window for the APC chemistry can be expanded to higher aqueous acidities (0.5 < p[H^+^] < 2.0) through structural modification of the chelator, targeting functional groups which increase the acidity of amine sites. Our recent structure–function relationship studies for aminopolycarboxylate complexants identified a 6-carboxypyridin-2-yl-methyl pendant arm, commonly known as *N*-2-methylpicolinate, as a promising substitutent to strengthen metal complexation in acidic media^15^. A replacement of a single acetate arm of ethylenediamine-*N*,*N*,*N*′,*N*′-tetraacetic acid, H_4_edta, by a picolinate moiety yielded H_4_edta-mpic (Fig. 1), which significantly increased the total ligand acidity (∑p*K*_a_), while maintaining the capacity for An^3+^/Ln^3+^ differentiation. Further symmetrical acetate-for-picolinate exchange on the H_4_edta molecular motif yields H_4_octapa, an octadentate acyclic aminopolycarboxylate reagent, recently demonstrated as a versatile radiometal chelator for radiopharmaceutical uses (Fig. 1)^16,17^. The *N*-2-methylpicolinate groups offer structural pre-organization to increase the stability of metal chelate and strong electron-inducing effects which increase the total acidity of the APC ligand. Accordingly, aqueous complexants such as H_4_octapa may sustain An^3+^/Ln^3+^ differentiation in aqueous mixtures of increased acidity. For H_4_edta-mpic, the *β*_101_(Am)/*β*_101_(Nd) ratio remained similar to that of H_4_edta. This would indicate that, despite the introduction of additional *N*-donor atom, no appreciable enhancement in 4*f*/5*f* differentiation should be expected for H_4_octapa upon the introduction of a second picolinate group.

An overall enhancement in ligand softness, which has been shown to play an important role in separation of trivalent An from Ln, can be attained through an introduction of additional nitrogen donor atoms into the APC structure. This study examines trivalent *f*-element complexation by a novel APC structure, H_4_pypa-peg, where a pyridine ring is added to H_4_octapa ligand as illustrated in Fig. 1. Both reagents are based on the ethylenediaminetetraacetatic acid structural motif, where, for H_4_octapa, two *N*-2-methylpicolinate groups replace two acetate pendant arms, and, for H_4_pypa-peg, a pyridine ring is also centrally added. The H_4_pypa-peg also includes a polyethylene glycol moiety to improve solubility in aqueous electrolyte mixtures. The structural modifications enable the study of chelate stability induced by the rigidity of the binding pocket, total ligand acidity and the electron-inducing effects on the soft nitrogen donor atoms for reagents containing 2-methylpicolinate pendant arms. A rigid, pre-organized binding pocket of H_4_octapa is compared with a more flexible H_4_pypa-peg, where a central pyridine also introduces additional nitrogen donor to seek enhanced preference for actinide complexation.

Trivalent actinide coordination with H_4_pypa-peg and H_4_octapa was studied to determine whether the insertion of the pyridine functionality in H_4_octapa structure yields a structural modification suitable for deployment in trivalent 4*f*/5*f* element separations. Potentiometric titrations were performed for both APC ligands to compare their protonation equilibria and interpret metal complexation results. The complexation of trivalent Ln and An (Am^3+^, Cm^3+^, and Cf^3+^) was characterized using potentiometry, spectrophotometry, and competitive solvent extraction and the interpretation of the acquired data was aided by trivalent *f*-element complexation studies for H_4_edta, H_5_dtpa, and triethylenetetraamine-*N,N,N',N'',N''',N'''*-hexaacetic acid (H_6_ttha) in 2.0 M (Na^+^/H^+^)ClO_4_ electrolyte medium.

## Methods

### Reagents

*Caution: All radiological manipulations were performed in a HEPA filtered fume hood or negative-pressure glovebox approved for radiological work.*

Preparation and standardization of Ln salts and electrolyte salts was reported previously^15,21,22^. Trivalent ^243^Am and ^248^Cm were retrieved from Idaho National Laboratory (INL) stocks, and purified using a diglycolic acid (DGA) extraction chromatographic resin (Eichrom), as described previously^15^. The DGA resin was also used to recycle trivalent ^249^Cf (sourced as the chloride salt from the Isotope Development and Production for Research and Applications Program through the Radiochemical Engineering and Development Center at Oak Ridge National Laboratory (ORNL) from previous experimental work). The ^243^Am(NO_3_)_3_ and ^248^Cm(ClO_4_)_3_ working stocks were prepared in 0.01 M HNO_3_ and HClO_4_, respectively, and standardized using UV/Visible spectroscopy^15,23–25^. Radiotracer mixtures of ^154^Eu^3+^ (Eckert & Ziegler) and ^243^Am^3+^, ^248^Cm^3+^ and ^249^Cf^3+^ for solvent extraction competitive studies were further diluted from stocks to ensure 300–500 Bq per 5 µL content. Bis(2-ethylhexyl)phosphoric acid (97%, Millipore-Sigma, HDEHP) was purified using the copper salt precipitation method^26,27^. Potentiometric titration indicated 99.7 ± 0.3% purity of the HDEHP extractant. Octane diluent (99 + %) was purchased from Millipore-Sigma and used without any further purification.

### Ligand synthesis

#### H_4_octapa

The ligand was synthesized using an experimental protocol reported by Platas-Iglesias et al.^28^ It was recrystallized from water twice prior to thermodynamic studies. The purity of the complexant was > 99% as verified using ^1^H NMR.

#### H_4_pypa-peg

6,6'-((((4-(1-(2-(2-(2-hydroxyethoxy)ethoxy)ethyl)-1H-1,2,3-triazol-4-yl)pyridine-2,6-diyl)bis–(methylene))bis–((carboxymethyl)azanediyl))bis–(methylene)) dipicolinic acid, H_4_pypa-peg, was prepared in five synthesis steps that are described in detail in the Supplemental Information. Final purity of product was > 99% as verified using ^1^H NMR and complexometric titration with a well-characterized ^243^Am stock solution.

### Potentiometric titrations

All potentiometric titrations were run using a Mettler Toledo T90 autotitrator equipped with an Orion Ross Semi-micro glass electrode with the junction stabilization solution changed to 5.0 M NaCl to prevent precipitation with perchlorate media. All titrations were performed under a hydrated nitrogen atmosphere. The forward titration measurements (acidic titrand, basic titrant) of H_4_octapa in nitrate electrolyte medium were performed as described previously due to the limited solubility of this ligand in a 2 M (Na^+^/H^+^)ClO_4_ medium^15^. Acid dissociation constants of H_4_octapa in perchlorate electrolyte medium were also determined by titrating in reverse (basic titrand, acidic titrant) to alleviate the solubility concerns. Titrations of H_4_pypa-peg were performed in reduced-volume glass titration cups (10 mL nominal) due to the limited supply of this ligand. All potentiometric titrations were run in triplicate. The choice of using p[H^+^] was explained in Heathman et al.^15^, and the calibration of the p[H^+^] scale utilized Gran analysis through titration of a strong acid with the base used in the potentiometric titrations^29–31^. Potentiometric titration data was modeled and analyzed using Hyperquad2013 to determine acid dissociation and metal complexation stability constants^32,33^.

### Spectroscopic studies

Changes in the optical absorption characteristics of Nd^3+^ were monitored using a Cary 6000i UV/Vis–NIR Spectrometer (Agilent) using 1 cm semimicro quartz cuvettes (Starna) in double-beam mode, where the ^4^I_9/2_ → ^4^G_5/2_, ^2^G_7/2_ transitions^34^ were monitored between 560 and 605 nm with a 0.075 nm interval and 1.0 nm spectral bandwidth. Changes in the optical absorption characteristics of ^243^Am^3+^ and ^248^Cm^3+^ were monitored using a Flame-S-VIS–NIR-ES spectrometer coupled to a DH-2000-BAL dual-mode light source (Ocean Optics) with a single 1 cm semimicro quartz cuvette (Starna). The light source and spectrometer were optically coupled to the CUV sample (Ocean Optics) holder using 2 m fiberoptics (200 μm) and the parameters for acquisition were 0.369 nm interval and 1000 scans averaged with an integration time/scan 1.08 ms (total acquisition time ~ 1 s). The complexation of Am^3+^ with the ligands was monitored in the 495–525 nm spectral region, looking at the ^7^*F*_0_′ → ^5^*L*_6_′ transition^23,35^. For Cm^3+^, ligand complexation was monitored in the 365–410 nm spectral region, looking at the ^*8*^*S*_*7/2*_ → ^*2*^*J*_*i*_ (*i* = {15}, {13,9}, {11,17}) manifold transitions^24,25,36^. Changes in aqueous p[H^+^] were monitored throughout each titration step using a Gran-calibrated Orion Ross Semi-micro glass electrode. Baseline correction was applied to all datasets and was typically a simple linear subtraction. However, in the case of the ^248^Cm/H_4_pypa-peg spectra, the tail end of the ligand absorption overlapped the hypersensitive Cm peaks. In this case the background correction was applied using a 5th-order empirical polynomial. Finally, the spectroscopic data was analyzed using the HypSpec software package^33,37^. Full details of the titrand and titrant solutions for each titration are included in the Supplemental Information.

Fluorescence measurements used a HORIBA Jobin Yvon IBH FluoroLog-3 fluorometer adapted for time-resolved studies. A submicrosecond xenon flash lamp (Jobin Yvon, 5000XeF) was used as a light source. The DAS 6 software (HORIBA Jobin Yvon IBH) was used for decay analysis and data fitting. Single and double exponential decay curves were used to model the observed luminescence lifetime data yielding *χ*^2^ values ranging between 1.01 and 1.08.

### Phase distribution measurements

Stability constants for the coordination of Eu^3+^, Am^3+^, Cm^3+^, and Cf^3+^ were also achieved by monitoring the suppression of liquid–liquid partitioning of the radiotracer due to the increasing presence of an aqueous complexant. Aqueous solutions were prepared containing sub-millimolar amounts of ligand while maintaining p[H^+^] at 1.7, 1.8, 1.9, 2.0, and 2.1. Ionic strength was maintained at 2.00 M using sodium perchlorate. Aqueous complexation was balanced with the appropriate choice of HDEHP concentration in octane to allow accurate quantification of metal distribution. Non-aqueous phases were pre-equilibrated three times with 2.00 M NaClO_4_ at the corresponding p[H^+^]. Preliminary time-dependent studies indicated the phase transfer equilibrium was attained for all measurements with H_4_octapa and H_4_pypa-peg (Figures S2 and S3 in the SI). For the mixtures traced with radioisotopes, the activity was measured using gamma spectroscopy (ORTEC GEM50P4 coaxial HPGe detector, DSPEC gamma spectrometer) for ^154^Eu (123.07 keV), ^243^Am (74.66 keV), and ^249^Cf (388.17 keV) and liquid scintillation counting (Perkin Elmer Tri-Carb 3180 TR/SL) for ^248^Cm. The ratio of radioisotope activity in the organic and aqueous phases defined the liquid–liquid distribution.

### Computational methods and models

Density functional theory (DFT) calculations using Gaussian 16^38^ have been performed to identify the most stable structures of the Eu^3+^ complexes with the H_4_octapa and H_4_pypa ligands containing two and one water molecules, respectively (see the SI section for details). Each complex was placed in a periodic cubic box of 15.226 Å (0.47 mol/L) and explicitly solvated with ≥ 85 water molecules. The initial configurations for AIMD simulations were generated by the MedeA Amorphous Cell Builder^39^ and preequilibrated for AIMD using the PCFF + ^40^ force field supported in MedeA-LAMMPS^39,41^. These simulations were carried out in an NVT ensemble at a temperature of 300 K for 5 ns, in which only the solvent molecules were allowed to equilibrate.

After initial equilibration of the [Eu(octapa)(H_2_O)_2_]^−^ and [Eu(pypa)(H_2_O)]^−^ complexes with explicit water molecules, first-principles molecular dynamics (MD) simulations based on density functional theory (DFT) were carried out in the Born–Oppenheimer approximation using the VASP software^42–45^. The valence electronic states were expanded in a basis of plane waves. The core-valence interaction was described using the Projector Augmented Wave (PAW) approach^46,47^. All ab initio MD (AIMD) calculations utilized a plane wave kinetic energy cutoff of 400 eV and the $\Gamma$ point approximation. The PBE GGA functional^48,49^ was used to describe the exchange–correlation energy together with the DFT-D3 method of Grimme^50^ for dispersion interactions. The Self-Consistent Field (SCF) convergence threshold was set to 10^−4^ eV in all the calculations. During the SCF solution, a Pulay scheme^51^, as implemented in VASP was used for charge density mixing. The trivalent Eu was modeled using the large core (LC) pseudopotential with six *f* electrons placed in the core. This is justified, because the Eu 4*f* orbitals typically have a negligible overlap with the frontier orbitals responsible for the Eu-ligand bonding^52^, whereas the placement of electrons in the valence shell can lead to spurious delocalization of localized *f* electrons. The employed method was shown to provide reliable Eu-EDTA bond distances in the solid state^15^, justifying it for predicting the solution structure of Eu^3+^ complexes.

For [Eu(octapa)(H_2_O)_2_]^−^ and [Eu(pypa)(H_2_O)]^−^ complexes, the initial configurations thus obtained were subjected to short geometry optimization using VASP, followed by AIMD simulations with a timestep of 1.0 fs. The Nosé-Hoover thermostat was adopted to maintain a temperature of 300 K for the complex with [octapa]^4−^, but the simulation temperature for the complex with [pypa]^4−^ was raised to 328 K to speed up the equilibration process. Additional AIMD simulations were performed for the [pypa]^4−^ complex at 300 K using the CP2K software^53^ to verify that the temperature variation has a minimal impact on the metal–ligand bond distances (Table S1). The–1 charge on the complex was compensated by a uniform background charge. We obtained ≥ 116 ps long trajectories, showing a transition of one of the water molecules from the inner to the outer shell at ~ 70 ps in both cases. Two windows of the last 40 ps for two different coordination geometries were chosen for structural analysis. Graphical visualization of the solvated structure was prepared using VMD^54^ and the analysis of the trajectory was performed using GROMACS^55^. The coordination number, CN(t), was defined by Eq. (1), as follows^56^,

1 $$CN\left( t \right) = \mathop \sum \limits_{i = 1}^{Natom} \frac{{1 - \left( {\frac{{r_{i} \left( t \right)}}{{r^{\dag } }}} \right)^{12} }}{{1 - \left( {\frac{{r_{i} \left( t \right)}}{{r^{\dag } }}} \right)^{24} }}$$

where ${r}_{i}$ is the distance of the *i*-th atom (oxygen or nitrogen atom) from Eu^3+^ and *r*^†^ is the location the first minimum appearing after the first Eu^3+^–O or Eu^3+^–N radial distribution function peak, as shown in Figure S5.

## Results and discussion

### Synthesis of Complexant with Improved Aqueous Solubility

**Figure 2:**
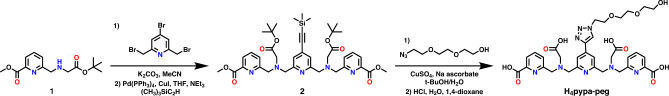
Synthesis of new H_4_pypa-peg complexant with improved aqueous solubility.

H_4_pypa features a centrally located pyridine, increasing the donor group count by one compared to H_4_octapa. However, compared to H_4_octapa, which has only two pyridine units, H_4_pypa's hydrophilicity is reduced due to the higher number of aromatic heterocycles in its structure. Nonetheless, the central pyridine serves as a further functionalization platform, as sucessfully demonstrated with the bioconjugation studies in nuclear medicine applications^18–20^. A more practical approach was devised to functionalize H_4_pypa to enhance its solubility in moderately acidic aqueous electrolyte mixtures (Fig. 2). The synthetic scheme incorporates a reactive site in the central pyridine fragment early on, enabling the Pd-catalyzed Sonogashira coupling reaction to introduce a further functionalizable alkyne moiety. Intermediate **2** is highly versatile as it can be utilized to append reactive or unreactive structural fragments for a variety of applications via Cu-catalyzed azide-alklyne cycloadition. To synthesize H_4_pypa-peg, **2** was reacted with 2-[2-(2-azidoethoxy)ethoxy]ethanol, followed by acid-catalyzed hydrolysis of methyl and *tert*-butyl esters. This simple structural modification resulted in significantly improved aqueous solubility of H_4_pypa-peg when compared to unfunctionalized H_4_pypa.

### Acid dissociation constants

**Figure 3:**
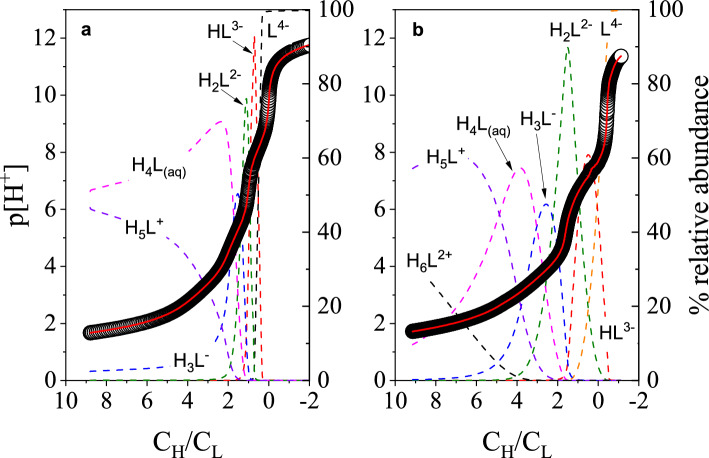
Potentiometric p[H^+^] trends collected for (**A**) H_4_octapa protonation titration at T = 25.0 ± 0.1 °C and I = 2.00 ± 0.01 M (Na^+^/H^+^)NO_3_, and (**B**) H_4_pypa-peg protonation titration at T = 25.0 ± 0.1 °C and I = 2.00 ± 0.01 M (Na^+^/H^+^)ClO_4_. (**A**) Titrand: V_initial_ = 25.012 mL, C_H__4octapa_ = 4.875 mM, C_H+_ = 0.043 M. Titrant: 0.397 M NaOH and 1.60 M NaNO_3_. (○) Experimental p[H+], (red solid line) calculated p[H^+^], (black dashed line) L^4−^, (red dashed line) HL^3−^, (green dashed line) H_2_L^2−^, (blue dashed line) H_3_L^−^, (pink dashed line) H_4_L_(Aq)_, (violet dashed line) H_5_L^+^. (**B**) Titrand: V_initial_ = 10.067 mL, C_H4__pypa-peg_ = 4.851 mM, C_H+_ = 0.044 M. Titrant: 0.125 M NaOH in 1.875 M NaClO_4_. (O) Experimental p[H^+^], (red solid line) calculated p[H^+^], (orange dashed line) L^4−^, (red dashed line) HL^3−^, (green dashed line) H_2_L^2−^, (blue dashed line) H_3_L^−^, (pink dashed line) H_4_L_(aq)_, (violet dashed line) H_5_L^+^, (black dashed line) H_6_L^2+^.

Changes in hydrogen ion concentration were monitored using a glass electrode for a solution of H_4_octapa as either NaOH or HClO_4_ were titrated into the solutions. Low solubility of H_4_octapa in an aqueous sodium perchlorate electrolyte medium prompted initial ligand titrations in a 2.0 M (Na^+^/H^+^)NO_3_ ionic background. The experimental potentiometric curve collected for a forward titration of H_4_octapa in (Na^+^/H^+^)NO_3_ is presented in Fig. 3A, alongside the distribution curves for the identified H_n_L species. Following those studies, the acid/base conditions were reversed and octapa^4−^ was titrated in 2.0 M (Na^+^/H^+^)ClO_4_ using HClO_4_ (SI Figure S7). The potentiometric curves are well respresented by five proton dissociation equilibria. The calculated equilibrium constants correspond with the protonation reactions for two aliphatic amines, two pyridine nitrogens, and one carboxylate group with the protonation sequence described by Eq. (2). The acid dissociation constants (p*K*_a_ =  − log_10_*K*_a_) for H_4_octapa are reported in Table 1 alongside previously reported values in the literature.

**Table 1:** Acid dissociation constants determined for H_4_octapa compared with those reported in literature^16,17^.

H_n_L	This work^a^	This work^b^	Ref. 16^c^	Ref. 17^d^
HL^3−^	8.16 (1)	8.41 (1)	8.52 (1)	8.58 (1)
H_2_L^2−^	5.27 (1)	5.57 (1)	5.40 (1)	5.43 (2)
H_3_L^−^	3.63 (1)	3.82 (1)	3.65 (1)	3.75 (1)
H_4_L_(aq)_	3.00 (1)	3.18 (1)	2.97 (1)	3.08 (1)
H_5_L^+^	1.63 (2)	1.63 (3)	1.66 (1)	2.21 (2)
H_6_L^2+^	–	–	–	1.61 (2)
H_7_L^3+^	–	–	–	0.12 (4)
H_8_L^4+^	–	–	–	− 0.46 (3)

^a^*I* = 2.0 M (Na^+^/H^+^)NO_3_ at 25.0 °C.

^b^*I* = 2.0 M (Na^+^/H^+^)ClO_4_ at 20.0 °C.

^c^*I* = 0.15 M NaCl at 25 °C.

^d^*I* = 0.16 M NaCl at 25 °C.

The initial three equilibrium reactions, (H_8_L^4+^, H_7_L^3+^, and H_6_L^2+^), occur in aqueous conditions too acidic for accurate quantification using a glass electrode. Those reactions correspond to the protonation reactions of carboxylate groups. The fourth acid dissociation equilibria (H_5_L^+^) may be attributed to the weakest carboxylic acid proton. The remaining p*K*_a_ sequence describes the protonation reactions of amine sites, with the initial two (H_4_L and H_3_L^−^) consistent with acidities of nitrogen within the pyridinecarboxylate structure, and the latter two (H_2_L^2−^ and HL^3−^) characteristic of the amine sites of the ethylenediamine backbone. Some differences between nitrate and perchlorate aqueous electrolyte media are noticeable, especially for protonation constants ascribed to the amine groups. Also, as expected, ionic strength influence on the acid dissociation constants is evident, when compared with acid dissociation constants reported by Kálmán et al.^16^ and Jaraquemada-Peláez et al.^17^ However, a strong electron-withdrawing influence of the *N*-2-methylpicolinate groups is evident, significantly decreasing the p*K*_a_ values of the amine nitrogens.

2 $${\text{H}}_{9 - a} {\text{L}}^{ + 5 - a} { \leftrightarrows }{\text{H}}_{8 - a} {\text{L}}^{ + 4 - a} + {\text{H}}^{ + } \;\;\;K_{a} = \frac{{\left[ {H^{ + } } \right] \cdot \left[ {{\text{H}}_{8 - a} {\text{L}}^{ + 4 - a} } \right]}}{{\left[ {{\text{H}}_{9 - a} {\text{L}}^{ + 5 - a} } \right]}}\;\;\;a = { 1} \ldots \;{8}$$

The experimental potentiometric titration curve and the distribution curves for the associated H_n_L species for H_4_pypa-peg are presented in Fig. 3B. The measured p*K*_a_ values for H_4_pypa-peg in 2.0 M (Na^+^/H^+^)ClO_4_ are summarized in Table 2. Fitting of the experimental curves showed convergence when six acid dissociation constants were included in the model. For comparison, the protonation equilibria of H_4_edta were also studied in 2.0 M (Na^+^/H^+^)ClO_4_. The p*K*_a_ values for H_4_edta are listed in Table 2 and collected potentiometric titration curves (forward and reverse) are presented in SI Figure S8. The protonation constants for similar ligands reported in literature (H_4_pypa^18^ and H_4_py4pa^57^) are also listed in Table 2 for comparison. For H_4_pypa-peg coordination pocket the protonation of H_9_L^5+^, H_7_L^3+^, and H_6_L^2+^ cannot be characterized using a glass electrode. The H_6_L^2+^ species likely represents a protonated carboxylate group, while H_5_L^+^ and H_4_L may describe the pyridinecarboxylate nitrogens. The equilibrium involving H_3_L^−^ likely describes the dissociation of the protonated pyridine nitrogen, while the two most basic species in this ligand (HL^3−^ and H_2_L^2−^) are likely the bridging amines in the H_4_pypa-peg backbone. Equation (3) describes the protonation sequence for H_4_pypa-peg.

3 $${\text{H}}_{10 - a} {\text{L}}^{ + 6 - a} { \leftrightarrows }{\text{H}}_{9 - a} {\text{L}}^{ + 5 - a} + {\text{H}}^{ + } \;\;\;K_{a} = \frac{{\left[ {H^{ + } } \right] \cdot \left[ {{\text{H}}_{9 - a} {\text{L}}^{ + 5 - a} } \right]}}{{\left[ {{\text{H}}_{10 - a} {\text{L}}^{ + 6 - a} } \right]}}\;\;\;a = { 1} \ldots {9}$$

**Table 2:** Acid dissociation constants determined for H_4_pypa-peg, H_4_edta and other structurally related APC ligands previously reported^18,57^.

H_n_L	H_4_pypa-peg^a^	H_4_edta^a^	H_4_pypa^b^	H_4_py4pa^c^
HL^3-^	7.44 (1)	8.71 (1)	7.78 (1)	6.96 (1)
H_2_L^2-^	6.45 (1)	6.23 (1)	6.78 (1)	6.07 (1)
H_3_L^-^	3.92 (1)	2.48 (3)	3.69 (1)	4.06 (2)
H_4_L_(aq)_	3.37 (1)	2.08 (1)	3.02 (1)	3.58 (2)
H_5_L^+^	2.45 (2)	–	2.23 (2)	2.71 (3)
H_6_L^2+^	1.43 (4)	–	2.06 (6)	2.55 (4)
H_7_L^3+^	–	–	1.70 (2)	2.31 (4)
H_8_L^4+^	–	–	− 0.37 (1)*	–
H_9_L^5+^	–	–	− 0.58 (2)*	–

^a^*I* = 2.0 M (Na^+^/H^+^)ClO_4_.

^b^From Ref. 18; *I* = 0.16 M NaCl and uncertainties reported to 1σ.

^c^From Ref. 57; *I* = 0.16 M NaCl and uncertainties reported to 1σ.

*Measured using UV/Vis spectroscopy.

Metal ion coordination by APC ligands in aqueous acidic mixtures is strongly dependent on the basicity of the dialkylamine sites. The sum of the acid dissociation constants for such amine sites, Σp*K*_a_(*N*), provides a relative comparison scale. This total dialkylamine basicity follows the H_4_edta > H_4_pypa-peg > H_4_octapa trend indicating that ethylenediamine backbones of H_4_pypa-peg and H_4_octapa are more acidic, relative to H_4_edta. The lowering of Σp*K*_a_(*N*) for both APC ligands may be attributed to the electron-withdrawing influence of the *N*-2-methylpicolinate groups, relative to *N*-acetate arms of H_4_edta. The strong inducing effects are lessened through the introduction of the pyridine group in the H_4_pypa-peg ligand.

### Lanthanide Complexation Behavior

**Figure 4:**
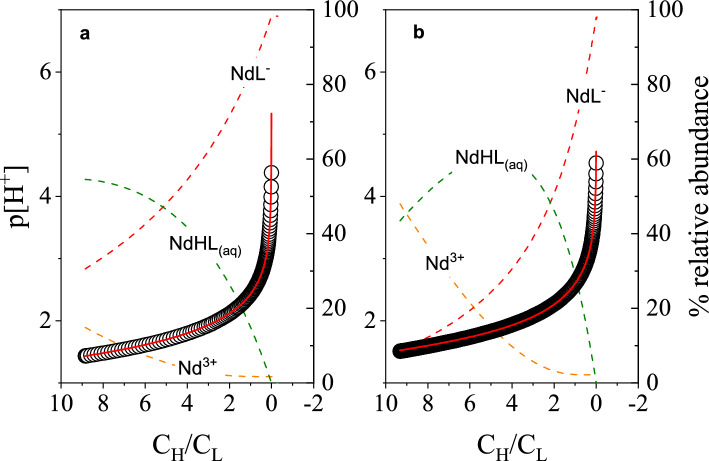
Potentiometric p[H^+^] trends collected for complexometric titrations between Nd^3+^ and (**A**) H_4_octapa at *T* = 25.0 ± 0.1 °C and *I* = 2.00 ± 0.01 M (Na^+^/H^+^) NO_3_ and (**B**) H_4_pypa-peg at *T* = 25.0 ± 0.1 °C and *I* = 2.00 ± 0.01 M (Na^+^/H^+^)ClO_4_. (**A**) Titrand: *V*_initial_ = 25.244 mL, *C*_H4octapa_ = 4.803 mM, *C*_H+_ = 0.043 M, *C*_Nd3+_ = 4.887 mM. Titrant: 0.397 M NaOH and 1.60 M NaNO_3_. (○) Experimental p[H^+^], (red solid line) calculated p[H^+^], (orange dashed line) Nd^3+^, (green dashed line) NdHL_(aq)_, (red dashed line) NdL^−^. (**B**) Titrand: V_initial_ = 10.153 mL, C_H4__pypa-peg_ = 4.700 mM, C_Nd3+_  = 4.841 mM, C_H+_  = 0.044 M. Titrant: 0.125 M NaOH in 1.875 M NaClO_4_. (○) Experimental p[H^+^], (red solid line) calculated p[H^+^], (orange dashed line) Nd^3+^, (green dashed line) NdHL_(aq)_, (red dashed line) NdL^−^.

The coordination of La^3+^, Nd^3+^, Eu^3+^, Tb^3+^, Dy^3+^, Ho^3+^, and Lu^3+^ with H_4_octapa and H_4_pypa-peg was studied. Figures 4A, B show the potentiometric curves collected when Nd^3+^ was titrated with H_4_octapa and H_4_pypa-peg, respectively. Both p[H^+^] curves show the characteristic buffering region due to deprotonation of metal–ligand complexes when titrated with base. The experimental p[H^+^] trend is best represented when considering the presence of MHL_(aq)_ and ML^–^ complexes as generalized by Eqs. (4) and (5). The progressive formation of the metal complexes throughout the titration is illustrated by species distribution curves, also shown in Figs. 4A, B. The complex formation equilibria are described by the conditional stability constants, *β*_101_ and *β*_111_, listed in Table 3.

**Table 3:** Conditional stability constants for the formation of ML^-^ and MHL_(aq)_ complexes for selected lanthanides (La^3+^, Nd^3+^, Eu^3+^, Tb^3+^, Dy^3+^, Ho^3+^, and Lu^3+^) for H_4_octapa, H_4_pypa-peg, and other structurally similar ligands.

Metal	H_4_octapa^a^			H_4_pypa-peg^a^			H_4_pypa^b^		H_4_py4pa^c^	
	log*β*_101_	log*β*_111_	log*K*_111_	log*β*_101_	log*β*_111_	log*K*_111_	log*β*_101_	log*K*_111_	log*β*_101_	log*K*_111_
La	17.77 (2)	19.30 (4)	1.53 (4)	17.86 (1)	20.18 (1)	2.32 (1)	19.54 (2)	3.24 (5)*	20.33 (3)^^^	3.78 (4)^^^
Nd	18.19 (1) 18.15 (1)^$^	19.92 (2) –	1.73 (2) –	18.20 (2) 18.15 (6)^&^	20.43 (2) 20.04 (4)^&^	2.22 (2) 1.89 (6)^&^	– –	– –	– –	– –
Eu	18.49 (2)	19.62 (4)	1.13 (4)	18.91 (1)	20.87 (1)	1.96 (1)	–	–	–	–
Tb	18.55 (2)	19.59 (7)	1.04 (7)	19.75 (2)	21.77 (2)	2.02 (2)	–	–	–	–
Dy	18.48 (2)	19.76 (1)	1.28 (2)	20.05 (1)	22.03 (2)	1.97 (2)	–	–	–	–
Ho	18.57 (2)	19.10 (1)	0.53 (2)	20.50 (4)	22.31 (4)	1.80 (4)	–	–	–	–
Lu	17.97 (3)	19.66 (4)	1.69 (4)	20.72 (5)	22.60 (5)	1.87 (5)	22.20 (2)	3.60 (6)*	–	–

^a^*I* = 2.0 M (Na^+^/H^+^)ClO_4_ and uncertainties reported to ± 3σ. *T* = 20.0 °C.

^b^From Ref.^18^; Potentiometric titrations with *I* = 0.16 M NaCl and uncertainties reported to 1σ.

^c^From Ref.^57^; uncertainties reported to 1σ.

^^^Obtained using ligand-ligand potentiometric competition with H_4_edta at 25 °C and 0.16 M NaCl.

^$^Spectrophotometrically determined in 2.0 M (Na^+^/H^+^)NO_3_.

^&^Spectrophotometrically determined in 2.0 M (Na^+^/H^+^)ClO_4_.

*From in-batch spectrophotometric competition at 25 °C, not evaluated at constant *I* = 0.16 M NaCl.

**Figure 5:**
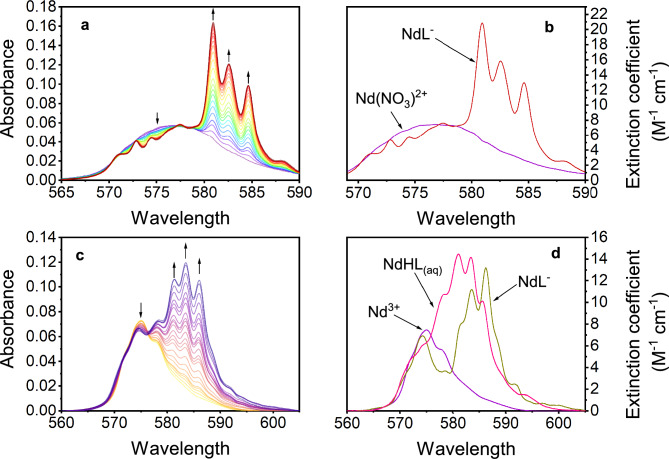
Changes in the optical absorption spectra for neodymium ion induced by (**A**) H_4_octapa in 2.00 M (Na^+^/H^+^)NO_3_ and (**C**) H_4_pypa-peg in 2.00 M (Na^+^/H^+^)ClO_4_ at *T* = 20 ± 1 °C. (**A**) Titrand conditions: *V*_initial_ = 0.809 mL, C_Nd3+_= 7.62 mM, p[H^+^]_initial_ = 1.40. Titrant conditions: *V*_titrant added_ = 0.529 mL, C_Nd3+_= 7.65 mM, C_H4octapa_ = 9.75 mM, p[H^+^] ≈ 6. (**C**) Titrand conditions: V_initial_ = 0.801 mL, C_Nd3+_  = 9.817 mM, p[H^+^] = 1.61. Titrant: C_H4__pypa-peg_ = 24.584 mM, C_Nd3+_  = 9.913 mM, p[H^+^] = 3.59. The calculated molar absorptivities (*ε*_A_)are shown in panels (**B**) for H_4_octapa species and (**D**) for H_4_pypa-peg species.

The coordination of Nd^3+^ by H_4_octapa and H_4_pypa-peg was also studied using spectroscopic methods. Figures 5A, B show the collected spectrophotometric signatures for H_4_octapa and H_4_pypa-peg, respectively.

4 $$M^{3 + } + L^{4 - } { \leftrightarrows }ML^{ - } \;\;\;\beta_{101} = \frac{{\left[ {ML^{ - } } \right]}}{{[M^{3 + } ]\left[ {L^{4 - } } \right]}}$$

5 $$M^{3 + } + H^{ + } + L^{4 - } { \leftrightarrows }MHL_{{\left( {aq} \right)}} \;\;\;\beta_{111} = \frac{{\left[ {MHL_{{\left( {aq} \right)}} } \right]}}{{[M^{3 + } \left] {\left[ {L^{4 - } } \right]} \right[H^{ + } ]}}$$

Optical absorption features of ^4^I_9/2_ → ^4^G_5/2_, ^2^G_7/2_ transitions for Nd^3+^ are perturbed due to the introduction of increasing quantities of complexants, resulting in characteristic red-shifted changes. In Fig. 5A a broad absorption band with λ_max_ = 577 nm is distinctly different to the optical absorption of free Nd^3+^ metal in Fig. 5B. The differences can be attributed to the nitrate complexation of Nd^3+^ as the spectrophotometric titration with H_4_octapa was performed in 2.0 M (Na^+^/H^+^)NO_3_. Analysis of the spectral data collected for the titration with H_4_octapa (Fig. 5C) suggests the presence of two uniquely absorbing metal species: a ${\text{NdNO}}_{3}^{{{2} + }}$ and NdL^−^, suggesting a quantitative displacement of the weak nitrate ligand by H_4_octapa. The HypSpec interpretation of the Nd/pypa-peg titration points to the presence of three absorbing species: Nd^3+^, NdHL_(aq)_, and NdL^−^. The calculated molar absorptivities for those species are shown in Fig. 5D. The conditional stability constants obtained spectroscopically are listed in Table 3, showing good agreement with the stability constants determined using potentiometry. The complex stability constants for structurally similar APC ligands found in the literature are also presented (H_4_pypa^18^ and H_4_py4pa^57^).

Relative to H_4_edta, both H_4_octapa and H_4_pypa-peg form complexes of increased stability, as the substitution of two *N*-acetate pendant arms by *N*-2-methylpicolinates increases the denticity of the coordination pocket. The H_4_octapa is a well-known octadentate chelator of trivalent *f*-elements, while, with the additional donor group, H_4_pypa-peg has the potential to displace all water molecules from the hydration zone of trivalent *f*-elements. The luminescence lifetime measurements of Eu^3+^ in presence of H_4_pypa-peg were performed to study the ligand-induced metal dehydration. The aqueous p[H^+^] = 1.54 and 4.70 were chosen for the measurements as conditional stability constants indicated high abundance of [Eu(Hpypa-peg)]_(aq)_ and $\left[ {{\text{Eu}}\left({{\text{pypa-peg}}} \right)} \right]_{{({\text{aq}})}}^{ - }$ species, respectively. An estimated 1:1 molar ratio of those metal complexes was also investigated at p[H^+^] = 1.99. The luminescence lifetime decay trends are shown in SI Figure S9, and the fitted decay constants (*τ*) and estimated inner-sphere hydration numbers (${\eta }_{\mathrm{H}2\mathrm{O}}$) for each complex are summarized in SI Table S2. The data suggests that at low p[H^+^], the [Eu(Hpypa-peg)]_(aq)_ complex is heptadentate with two inner-sphere waters, while at higher p[H^+^], the [Eu(pypa-peg)]^−^ complex is ocatadentate with one water molecule in the coordination sphere of Eu^3+^. The presence of single water molecule in the ML^−^ complex of H_4_pypa-peg may indicate that not all donor atoms of this chelator participate in metal ion complexation. The inspection of the acquired conditional stability constants may help with identifying this donor group.

The stability of the ML^−^ complexes for light lanthanides with H_4_pypa-peg shows remarkable similarity to H_4_octapa. This supports the luminescence finding, pointing to the same binding denticity for both APC ligands. The coordination pockets are well suited to accept the bulkier, less charge dense 4*f* cations. A sharp contrast is observed for the complexation of heavier lanthanides (Tb^3+^ through Lu^3+^) with H_4_octapa and H_4_pypa-peg. After initially increasing, the stability constant trend for H_4_octapa reaches a plateau and *β*_101_ constants for the complexation of Eu^3+^, Tb^3+^, Dy^3+^, and Ho^3+^ with H_4_octapa do not vary appreciably, and eventually decrease for Lu^3+^. This stability constant trend has been attributed to steric factors which increase when H_4_octapa coordinates heavier trivalent lanthanides^16,17^. Similar impact of steric hindrance on the stability constants for trivalent *f*-element complexation has been observed for large polydentate APC chelators such as H_5_dtpa and H_6_ttha^58,59^. The cross-lanthanide stability constant trend increases monotonically for H_4_pypa-peg, resembling H_4_edta. This trend is also observed with the non-functionalized H_4_pypa, showing a 1000-fold stability increase for ML^−^ complexes. Accordingly, the introduction of the central pyridine ring in H_4_pypa-peg lowers the rigidity of the coordination pocket of H_4_octapa. The MHL_(aq)_ complex for H_4_pypa-peg forms more readily (higher abundance) at higher p[H^+^] relative to H_4_octapa in accordance with its lower overall total ligand acidity.

### Structural analysis of the EuL^−^ complexes from ab initio molecular dynamics simulations

In order to further understand the structural properties of metal complexes with H_4_octapa and H_4_pypa-peg, we simulated the [Eu(octapa)(H_2_O)_2_]^−^ and [Eu(pypa)(H_2_O)]^−^ complex using AIMD. Figure 5 shows evolution of various bond distances and coordination numbers (Eq. (1)) over the course of the simulations. The data illustrate the interconversion dynamics between ten and nine-coordinate complexes, capturing the departure of one water molecule from the inner to the outer shell for both the Eu^3+^-octapa and Eu^3+^-pypa complexes. The snapshots of the Eu^3+^-octapa and Eu^3+^-pypa complexes in two coordination states (CN = 9 and 10) are shown in Figure S4. Structural correlations based on the radial distribution functions (RDFs) for [Eu(octapa)(H_2_O)_n_]^−^ (n = 1, 2) and [Eu(pypa)(H_2_O)_n_]^−^ (n = 0, 1) complexes are provided in Figure S5.

Table 4 provides a comparison of the Eu–O and Eu-N bond distances for Eu^3+^-octapa in the presence of either one or two inner-sphere water molecules and for Eu^3+^-pypa in the presence or absence of one inner-sphere water molecule. Analysis of the Eu–O and Eu–N bond lengths shows that the metal–ligand bond lengths are in most cases shortened in the nine-coordinate structure compared to those in the ten-coordinate structure. The stronger bond shortening going from CN = 10 to CN = 9 is observed for the acetate-COO^−^ groups that become largely indistinguishable from the pyridine-COO^−^ groups. At the same time, the bond distances to the amine N atoms remain substantially longer (by 0.10–0.18 Å) compared to pyridine N atoms, which can be attributed to higher donor ability and better preorganization of the latter. An exception to this is one terminal pyridine N_1_ atom that exhibits a substantial bond elongation in the ten-coordinate pypa complex. Likewise, the amine N_2_ atom adjacent to it shows the longest bond elongations and the largest bond fluctuation, providing flexibility to accommodate one inner-sphere water molecule.

**Table 4:** Bond distances (Å) in EuL^−^ complexes with H_4_octapa and H_4_pypa ligands from AIMD simulations. The last 40 ps segment for each coordination state was taken for structural analysis.

	[Eu(octapa)(H_2_O)_n_]^−^		[Eu(pypa)(H_2_O)_n_]^−^	
	n = 2	n = 1	n = 1	n = 0
Eu–O_**1-pyr**_	2.45 ± 0.10	2.45 ± 0.10	2.46 ± 0.10	2.43 ± 0.10
Eu–O_**2-acet**_	2.58 ± 0.17	2.43 ± 0.10	2.48 ± 0.11	2.42 ± 0.10
Eu–O_**3-acet**_	2.50 ± 0.13	2.39 ± 0.10	2.40 ± 0.10	2.40 ± 0.10
Eu–O_**4-pyr**_	2.45 ± 0.10	2.42 ± 0.10	2.52 ± 0.13	2.46 ± 0.10
Eu–O_**W1**_	2.59 ± 0.15	4.49 ± 0.25	2.52 ± 0.11	5.85 ± 0.68
Eu–O_**W2**_	2.52 ± 0.11	2.50 ± 0.10	–	–
Eu–N_**1-pyr**_	2.67 ± 0.10	2.66 ± 0.10	2.74 ± 0.10	2.64 ± 0.10
Eu–N_**2-amine**_	2.80 ± 0.10	2.85 ± 0.11	2.87 ± 0.12	2.78 ± 0.10
Eu–N_**3-amine**_	2.81 ± 0.10	2.74 ± 0.10	2.74 ± 0.10	2.77 ± 0.10
Eu–N_**4-pyr**_	2.67 ± 0.10	2.60 ± 0.10	2.65 ± 0.10	2.61 ± 0.10
Eu–N_**5-pyr**_	–	–	2.62 ± 0.10	2.59 ± 0.10

Based on luminescence and paramagnetic NMR measurements^28^, the Ln^3+^-octapa complexes in solution were inferred to be nine-coordinate, with one water molecule completing the primary coordination sphere. Unlike coordination environments in solution, the X-ray studies in the solid state for La^3+^^17^ and Gd^3+^^60^ showed a ten-coordinate geometry, where two water molecules reside in the primary shell. Differences between the solid- and solution-state interactions for lanthanide complexes that include crystal packing and outer-sphere solvent effects, respectively, are not uncommon, with the Eu^3+^-edta complex being another example^15,61^, where the number of water molecules increases from 2 in solution to 3 in the solid state. There is a relative paucity of knowledge pertaining to solution phase coordination that is hard to unambiguously probe experimentally. Thus, our DFT-based AIMD simulation was used to shed light into to (i) the evolution of the crystalline-like complex in aqueous environment and (ii) corroborate the hydration numbers from the luminescence lifetimes.

**Figure 6:**
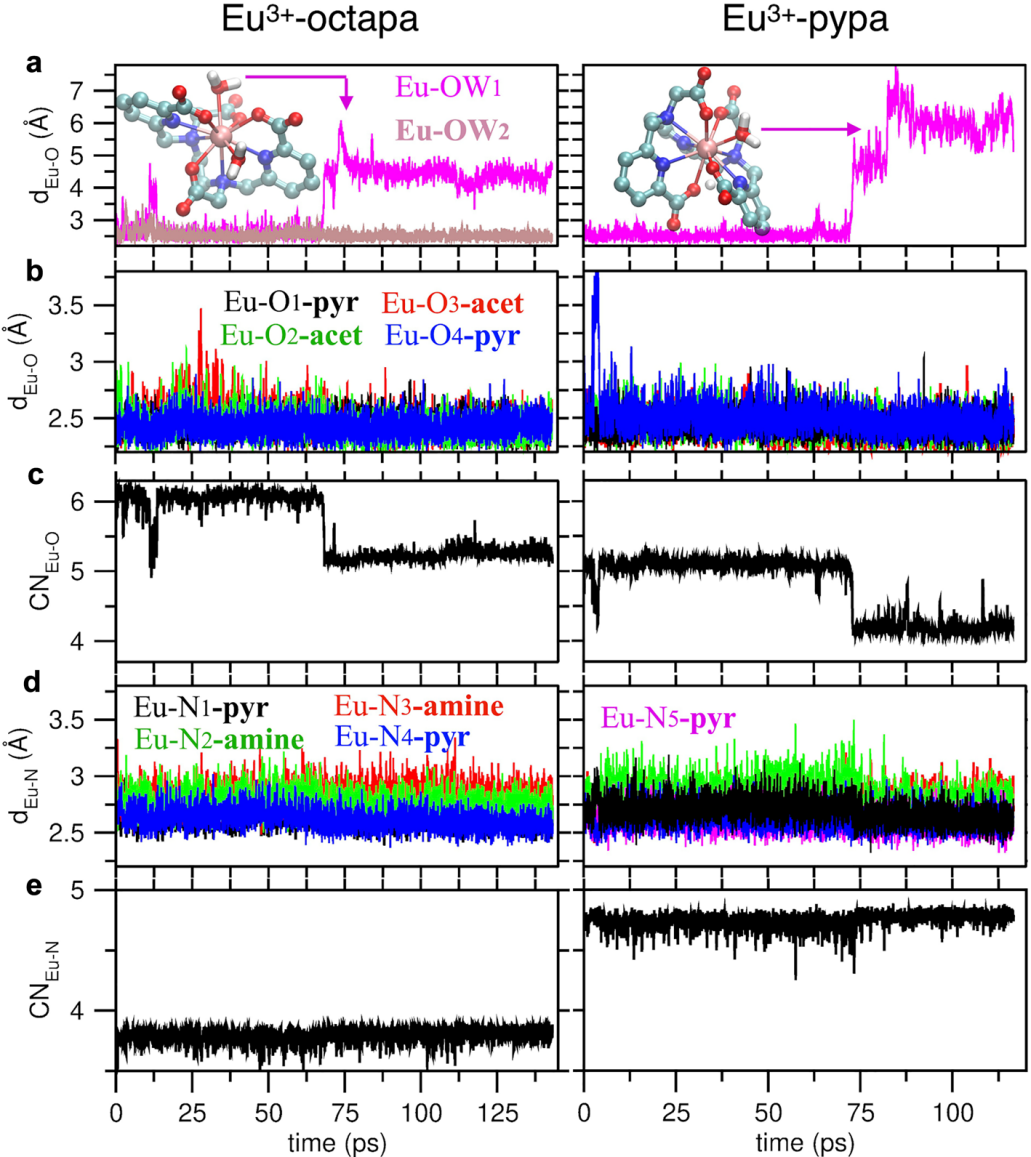
Time-dependent structural metrics for EuL^−^ complexes with H_4_octapa and H_4_pypa-peg ligands. (**A**) Fluctuation of the individual Eu^3+^–O bond distances, (**B**) oxygen coordination number of Eu^3+^ considering the Eu^3+^–O cutoff distance of 3.4 Å (based on the first minimum of the Eu^3+^–O RDF shown in Figure S5), (**C**) fluctuation of the individual Eu^3+^–N bond distances, and (**D**) nitrogen coordination number of Eu^3+^ considering the Eu^3+^–N cutoff distance of 3.5 Å (based on the first minimum of the Eu^3+^–N RDF shown in Figure S5). Structures of ten-coordinated complexes are shown in the inset. The middle pyridine nitrogen in pypa-peg is labeled as N_5-pyr_.

Two unsuccessful departure attempts for one of the inner-sphere water molecules in [Eu(octapa)(H_2_O)_2_]^−^ were observed during the first 15 ps of the AIMD simulation. This transition finally succeeded at ~ 70 ps and the resulting [Eu(octapa)(H_2_O)]^−^ complex remained stable during the rest of the simulation (Fig. 6). Thus, in bulk water, according to the present MD simulation results, the interaction of the second water with the metal ion center is less favorable than the water-water interaction, which agrees with the presence of one coordinating water molecule based on luminescence studies.

An unsuccessful attempt for one of the pyridine-COO^−^ groups in [Eu(pypa)(H_2_O)]^−^ to depart the inner coordination shell was observed during the first 5 ps of the AIMD run. At around 70 ps the inner sphere water molecule moved to the second shell, rendering the complex nine-coordinate. However, unlike the octapa complex, the CN plot (Fig. 6B) shows several attempts for another water molecule to approach the Eu^3+^ center as close as 3.5 Å. It is hypothesized that longer AIMD simulations could result in a fully coordinated complex. This conjecture is based on the following two arguments. First, in the EuL^−^ complex with pypa, a large portion of the space around Eu^3+^ remains unobstructed by the ligand, potentially allowing one water molecule in and out of the inner shell. Second, AIMD simulations using the CP2K software package at 298.15 K (Fig. 5) indicate that the potential energies for CN = 10 and CN = 9 states are highly overlapping, which are essentially the same within the statistical uncertainty. This suggests a possibility for a stronger stabilization of the CN = 10 at room temperature compared to the elevated temperature (T = 328 K) at which VASP simulations were run. Interestingly, the water remains fully coordinated in the [Eu(pypa)(H_2_O)]^−^ complex in the AIMD simulations performed at T = 298.15 K, which passed the time threshold for water dissociated in the AIMD simulations performed at T = 328 K.

Finally, cluster calculations with implicit solvation were utilized to check which functional group is more likely to be protonated in the [Eu(Hpypa)(H_2_O)] complex. The results suggested a strong energetics preference (by at least 5 kcal/mol) for protonating one of the carboxylic groups attached to the amine over the protonation of the terminal pyridine nitrogen and significantly larger energy penalty for protonating the amine nitrogen (by more than 10 kcal/mol). This is consistent with the pK_a_ values of the functional groups present in the ligands.

### Actinide complexation behavior

Liquid–liquid partitioning of trivalent *f*-elements was monitored in presence of H_4_octapa and H_4_pypa-peg using a strong liquid cation exchanger, bis–(2-ethylhexyl)phosphoric acid, HDEHP. This competitive solvent extraction methodology was used to determine the conditional stability constants for complexation of Eu^3+^, Am^3+^, Cm^3+^, and Cf^3+^ with H_4_octapa and H_4_pypa-peg. The metals, present at radiotracer concentrations (>1 μM), distributed between an organic phase containing HDEHP in *n*-octane and an aqueous phase containing the complexant at varying concentrations and aqueous acidity. The interpretation of the competitive complexation liquid–liquid distribution data (SI Tables S3 through S5 for H_4_octapa and S6 through S8 for H_4_pypa-peg) considered two complex formation equilibria as described by Eqs. (4) and (5). All collected distribution data indicated the average ligand-to-metal stoichiometry of 1.00 ± 0.04 for H_4_octapa and 1.00 ± 0.03 for H_4_pypa-peg (see SI Figures S10A and S11A). The partitioning of a trivalent metal ion as facilitated by a dimerized HDEHP cation exchanger can be expressed by Eq. (6) as quantified by the extraction constant, *K*_ex_ (subscript *org* refers to organic-soluble species).

6 $$M^{3 + } + 3\left( {HDEHP} \right)_{2,org} { \leftrightarrows }M\left( {H\left( {DEHP} \right)_{2} } \right)_{3,org} + 3H^{ + } \;\;\;K_{ex} = \frac{{\left[ {M\left( {H\left( {DEHP} \right)_{2} } \right)_{3,org} } \right]\left[ {H^{ + } } \right]^{3} }}{{\left[ {M^{3 + } } \right]\left[ {\left( {HDEHP} \right)_{2,org} } \right]^{3} }}$$

The stoichiometry for the extraction of trivalent metal ion by HDEHP was verified at 2.85 ± 0.03 by slope analysis study using ^249^Cf^3+^ as representative ion for H_4_octapa (SI Figure S10B) and 2.78 ± 0.13 for H_4_pypa-peg (SI Figure S11B). The distribution of the metal in this competitive complexation liquid–liquid environment can be described by Eq. (7).

7 $$D_{M} = \left( {\left[ {M\left( {H\left( {DEHP} \right)_{2} } \right)_{3} } \right]_{org} } \right)/\left( {\left[ {M^{3 + } } \right] + \left[ {ML^{ - } } \right] + \left[ {\left( {MHL} \right)_{aq} } \right]} \right)$$

Substitution of equilibrium and extraction constants Eqs. (3)–(5) into Eq. (6) yields Eq. (8).

8 $$D_{M} = \left( {K_{ex} \left[ {\left( {HDEHP} \right)_{2,org} } \right]^{3} \left[ {H^{ + } } \right]^{3} } \right)/\left( {1 + \beta_{101} \left[ {L^{4 - } } \right] + \beta_{111} \left[ {L^{4 - } } \right]\left[ {H^{ + } } \right]} \right)$$

At constant HDEHP concentration and p[H^+^], when the distribution of the metal ion in absence of ligand is labelled as D^0^, the liquid–liquid partitioning of the metal will adhere to Eq. (9),

9 $$\frac{{D^{0} }}{{D_{M} }} - 1 = \beta^{app} \left[ {L^{4 - } } \right]$$

where *β*^app^ is the apparent stability constant as represented by *β* ^app^ = *β*_101_ + *β*_111_[H^+^]. Accordingly, when metal distribution is plotted as a function of free ligand concentration, the presence of ML^−^ and MHL_(aq)_ is revealed if the experimental data shows variance with aqueous p[H^+^].

**Figure 7:**
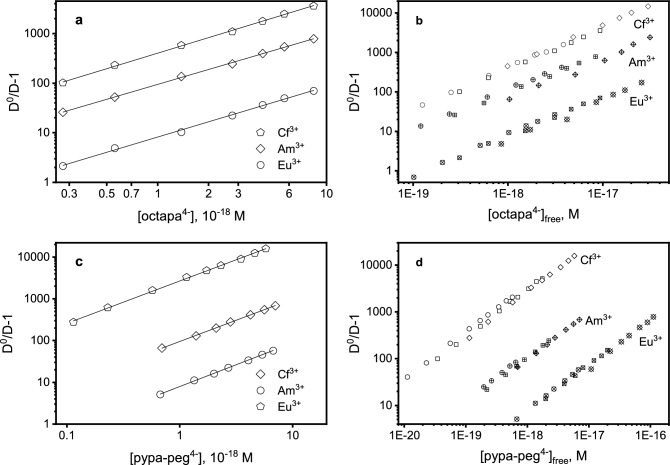
(**A**) *β*^app^ trends determined for the complexation of Cf ^3+^ (p[H^+^] 1.88), Am^3+^ (p[H^+^] 1.87) and Eu^3+^ (p[H^+^] 1.87) by octapa^4−^. Distribution ratio data is listed in Tables S3, S4, S5 in Supplemental Information. (**B**) p[H^+^]-dependence of *β*^app^ trends describing trivalent *f*-element coordination by H_4_octapa. Symbol legend: circles represent distribution data at p[H^+^] 1.80 (Cf), 1.80 (Am), 1.78 (Eu), squares at p[H^+^] 1.89 (Cf), 1.88 (Am), 1.88 (Eu), diamonds at p[H^+^] 2.01 (Cf), 2.02 (Am), 1.99 (Eu). (**C**) *β*^app^ trends determined for the complexation of Eu^3+^ (p[H^+^] = 2.00), Am^3+^ (p[H^+^] = 2.00), and Cf ^3+^ (p[H^+^] = 1.98) by pypa-peg^4−^. Distribution ratio data is listed in Tables S6, S7, S8 in the Supplemental Information. (**D**) p[H^+^]-dependence of *β*^app^ trends describing trivalent *f*-element coordination by pypa-peg^4−^. Symbol legend: circles represent distribution data at p[H^+^] = 2.00 (Eu), 1.79 (Am), 1.78 (Cf); squares at p[H^+^] = 2.09 (Eu), 1.90 (Am), 1.88 (Cf); diamonds at p[H^+^] = 2.25 (Eu), 2.00 (Am), 1.98 (Cf).

The Figs. 7A for H_4_octapa and Fig. 7C for H_4_pypa-peg show representative Eu^3+^, Am^3+^, and Cf^3+^ sets of dependencies collected at constant aqueous acidities to determine *β*^app^ constants from the slopes of the error-weighted least-squares regression analyses. Equivalent dependencies were also collected at different p[H^+^] conditions to study variation of *β*^app^ trends with p[H^+^] as presented in Figs. 7B,D for H_4_octapa and H_4_pypa-peg, respectively. The distribution plots for Eu^3+^, Am^3+^, and Cf^3+^ for H_4_pypa-peg clearly show variation with the changing aqueous acidity, which yielded good estimates for the conditional stability constants for the formation of ML^−^ and MHL_(aq)_ (Table 5). The collected trends for Cf^3+^ in the presence of H_4_octapa vary to a lesser extent, enabling the determination of *β*_101_ only. The p[H^+^] variation was not monitored for the coordination of ^248^Cm^3+^ with both APC ligands due to limited availability of this radioisotope. Accordingly, only *β*_101_ values are reported in Table 5 for the complexation of Cm^3+^ with H_4_octapa and H_4_pypa-peg. Good overall agreement between the stability constants acquired using solvent extraction and potentiometry was observed for the complexation of Eu^3+^.

**Table 5:** Conditional stability constants for trivalent actinides measured spectrophotometrically and through competitive metal complexation in liquid–liquid extractions.

Metal	H_4_octapa			H_4_pypa-peg		
	log*β*_101_	log*β*_111_	log*K*_111_	log*β*_101_	log*β*_111_	log*K*_111_
Eu^3+^	18.49 (2)* 18.59 (2)^^^	19.62 (4)* 20.45 (2)^^^	1.13 (4)* 1.86 (2)^^^	18.91 (1)* 18.7 (2)^^^	20.87 (1)* 20.6 (4)^^^	1.96 (1)* 1.9 (4)^^^
Am^3+^	19.28 (1)^&^ 19.3 (6)^^^	– 21.8 (2)^^^	– 2.5 (6)^^^	19.6 (1)^^^	21.8 (1)^^^	2.2 (1)^^^
Cm^3+^	19.4^^^	–	–	20.37 (2)^&^ 19.8^^^	– –	– –
Cf^3+^	20.6 (2)^^^	–	–	21.0 (4)^^^	23.2 (2)^^^	2.2 (1)^^^

^&^Spectrophotometrically measured, *I* = 2.0 M (Na^+^/H^+^)NO_3_ {H_4_octapa} or *I* = 2.0 M (Na^+^/H^+^)ClO_4_ {H_4_pypa-peg}, *T* = 25 ± 1 °C.

^^^Competitive solvent extractions, *T* = 20 ± 1 °C.

All errors reported to ± 3σ.

*Potentiometrically measured, *I* = 2.0 M (Na^+^/H^+^)ClO_4_, *T* = 20.0 ± 0.1 °C.

**Figure 8:**
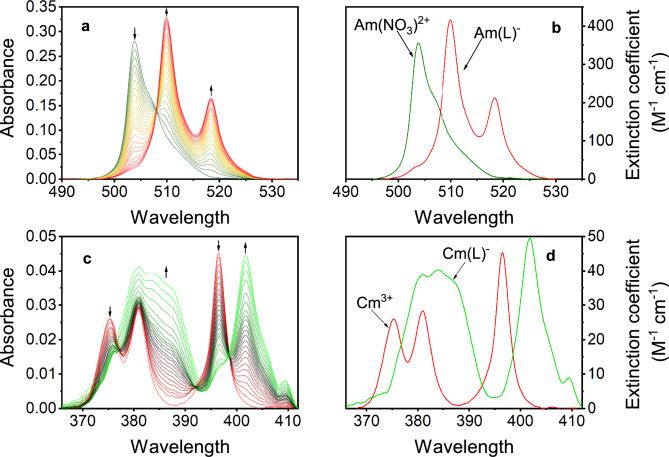
(**A**) Spectrophotometric titration of Am^3+^ with H_4_octapa. Experiment was conducted at 20 ± 1 °C with *I* = 2.00 M (Na^+^/H^+^)NO_3_. Titrand conditions: *V*_initial_ = 0.822 mL,C_Am3+_ = 0.788 mM, p[H^+^]_initial_ = 1.34. Titrant conditions: *V*_titrant added_ = 0.655 mL, C_Am3+_= 0.791 mM, C_H4octapa_ = 4.93 mM, p[H^+^] ≈ 7. (**B**) Calculated molar absorptivities (*ε*_A_) of the two fitted species in solution, Am^3+^ and [Am(octapa)]^−^. (**C**) Spectrophotometric titration of Cm^3+^ with H_4_pypa-peg. Experiment was conducted at 20 ± 1 °C with *I* = 2.00 M (Na^+^/H^+^)ClO_4_. Titrand: *V*_initial_ = 0.809 mL, C_Cm3+_  = 1.000 mM, p[H^+^] = 1.34. Titrant: C_H4pypa-peg_ = 15.098 mM, C_Cm3+_ = 1.002 mM, p[H^+^] ≈ 5–6. (**D**) Calculated molar absorptivities (*ε*_A_) of the two fitted species in solution, Cm^3+^ and [Cm(pypa-peg)]^−^.

To further validate the competitive solvent extraction methodology the complexation of Am^3+^ with H_4_octapa and Cm^3+^ with H_4_pypa-peg was studied spectrophotometrically. These titrations monitored the ligand-induced changes on the optical absorption spectra of free Am^3+^ at λ_max_ = 503.0 nm, (Fig. 8A) and Cm^3+^ at λ_max_ = 375.0, 380.6, 396.5 nm, (Fig. 8C). The presence of both ligands induced new red-shifted absorption signatures in each monitored titration. For H_4_octapa, Fig. 8B shows two light absorbing metal species were observed, assigned to Am^3+^/AmNO_3_^2+^ composite and [Am(octapa)]^−^ complex. Figure 8D shows the calculated molar absorptivity spectra for H_4_pypa-peg/Cm^3+^ spectrophotometric titration, where the presence of free Cm^3+^ and the ML^−^ complex were also observed. The calculated conditional stability constants are listed in Table 5. Excellent agreement between the solvent extraction and spectrophotometry was attained for the *β*_101_ values for the formation of [Am(octapa)]^−^ albeit the uncertainty associated with a constant measured using competitive liquid–liquid distribution is rather large and points out the shortcomings of this method. A variance between two analytical methods is larger for [Cm(pypa-peg)]^−^ possibly due to free ligand absorption interferences.

As observed for the trivalent 4*f* elements, the insertion of a central pyridine ring in the H_4_pypa-peg structure decreases the rigidity of the coordination pocket of H_4_octapa. Similar observation is noted for the trivalent 5*f* elements as the stability constants follow the Am^3+^ < Cm^3+^ < Cf^3+^ trend. This increase is also preserved in the case of H_4_octapa, which suggests that complexation of Cf^3+^ with this rigid ligand adheres to the trend followed by the light members of the *f*-elemental series. This increase is in accordance with the studies by H_5_dtpa Brandau et al. and Leguay et al.^62,63^ Accordingly, as in the case of bulky H_5_dtpa, the steric factors are not manifested for the complexation of Cf^3+^ with H_4_octapa.

**Figure 9:**
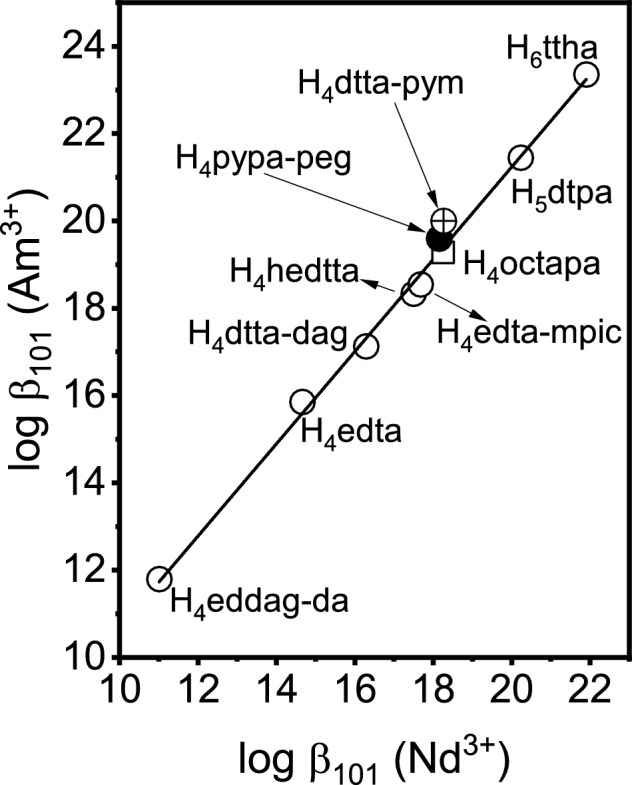
Am^3+^/Nd^3+^ stability constant ratio for H_4_pypa-peg (●) and H_4_octapa (□) as compared with those established for conventional APC reagents containing aminoacetate blocks only, and APC reagents containing *N*-2-pyridinylmethyl groups. The linear free energy relationship diagram was built by combining previously reported stability constants for Am^3+^ and Nd^3+^ complexation with ethylenediamine-*N*,*N*′-di(acetylglycine)-*N*,*N'*-diacetate, H_4_eddag-dag^39^, diethylenetriamine-*N*,*N''*-di(acetylglycine)-*N*,*N'*,*N''*-triacetate, H_5_dtta-dag^40^, *N*-(hydroxyethyl)-diethylenetriamine-*N*,*N*′,*N''*,*N''*-tetracetate), H_4_hedtta,^41^ and ethylenediamine-*N*-(2-methylpicolicate)-*N*,*N'*,*N'*-triacetic acid, H_4_edta-mpic^10^, and stability constants for Am^3+^ and Nd^3+^ complexation with ethylenediamine-*N*,*N*,*N'*,*N'*-tetraacetic acid, H_4_edta, diethylenetriamine-*N*,*N*,*N'*,*N''*,*N''*-pentaacetic acid, H_5_dtpa, and triethylenetetraamine-*N*,*N*,*N'*,*N''*,*N'''*,*N'''*-hexaacetic acid, H_6_ttha, redetermined in 2.0 M (Na^+^/H^+^)ClO_4_. For comparison, data for *N*-2-pyridylmethyl-diethylenetriamine-*N*,*N*′,*N*′′,*N*′′-tetraacetic acid, H_5_dtta-pym ( ⊕)^17^, is also included as another APC reagent containing a pyridine ring.

The effect of a fifth nitrogen donor atom in H_4_pypa-peg on the coordination of trivalent actinides can be evaluated through a comparison of metal complexation equilibria for trivalent 4*f* and 5*f* elements of similar charge density. Similar stabilities of metal complexes of Am^3+^ and analogous complexes with Nd^3+^ or Sm^3+^ are expected when complexing agents consist solely of oxygen donor atoms are expected^8^. The presence of donor atoms softer than oxygen, i.e. nitrogen or sulfur, is typically manifested by a steeper slope when the stabilities of 1:1 or 1:2 metal–ligand chelates of Am^3+^ versus Nd^3+^ and Sm^3+^ ions are co-related^8,59^. Figure 9 shows this linear free energy relationship for APC reagents containing aminoacetate blocks only. For consistency, stability constants used to establish this trend have been determined in 2.0 M (Na^+^/H^+^)ClO_4_^15,64–66^. The stability constants for Am^3+^ and Nd^3+^ complexation with H_5_dtpa and H_6_ttha, were also examined in 2.0 M (Na^+^/H^+^)ClO_4_ in a series of potentiometric and spectroscopic measurements to broaden the consistency of APC data presented in Fig. 9. For each APC reagent, acid dissociation constants (SI Table S9) and stability constants for coordination of Nd^3+^ and Am^3+^ (SI Table S10). The spectrophotometric titration plots are also provided for Nd^3+^ and Am^3+^ with H_4_edta (SI Figures S12 and S13), with H_5_dtpa (SI Figures S14 and S15) and with H_6_ttha (SI Figures S16 and S17). The slope of 1.06 for the linear free energy relationship constructed in Fig. 9 indicates the stability of the AmL complex is approximately 6% higher, relative to NdL, for the considered APC ligands. Previous studies of APC chelators containing *N-*2-pyridylmethyl groups have found the Am^3+^/Nd^3+^ stability constant ratios are even higher, finding departures from this linear free energy trend^22,67,68^. This is demonstrated for H_4_dtta-pym, where a single *N*-acetate group of H_5_dtpa was replaced by *N*-2-pyridylmethyl group^22^. This reagent does not adhere to the linear trend established in Fig. 9 and the upward deviation suggests that trivalent actinide binding strengthens with the addition of *sp*^2^-hybridized nitrogens^22^. In contrast, the presence of a pyridine ring in APC reagents containing 2-methylpicolinate functionality does not increase the preference for trivalent actinide binding as evidenced for H_4_edta-mpic^15^. This may be explained by the strong electron-withdrawing influence imposed on the pyridine nitrogen, depleting its electron density and rendering it similar to *sp*^3^-hybridized amine groups. These observations are also preserved for H_4_octapa, which shows no departure from the free energy relationship, and H_4_pypa-peg where only a small increase in the stability constant ratio for the complexation of Am^3+^ and Nd^3+^ is found. Figure 9 highlights both reagents to emphasize this. The participation of the central pyridine nitrogen in metal binding may be inferred from the enhanced differentiation of Am^3+^/Nd^3+^ pair. However, a lower *β*_101_ ratio, relative to H_4_dtta-pym, suggests that strong electron-inducing forces impact the softness of the central pyridinyl nitrogen of H_4_pypa-peg.

## Conclusions

The coordination studies of trivalent Ln and An by APC radiometal chelators H_4_octapa and H_4_pypa-peg show an intricate balance between the rigidity of the binding pocket, total ligand acidity, and the electron-inducing effects on the soft nitrogen donor atoms for reagents containing 2-methylpicolinate pendant arms. The 6-carboxypyridine-2-yl-methyl substituents increase the nitrogen acidity on the aminoacetate APC backbone, substantially lowering the operational pH window for efficient metal coordination. This structural modification comes at a cost of reduced rigidity of a binding pocket and aqueous solubility as demonstrated by the H_4_octapa. Steric hindrance, observed in the coordination of trivalent *f*-elements by H_4_octapa, reduces the chelate stability variance across the 4*f* series, similar to a bulky H_6_ttha, and in contrast to the trends observed for conventional APC reagents H_4_edta and H_5_dtpa. The inhibited rotational flexibility of H_4_octapa can be overcome with the addition of a central pyridine ring of H_4_pypa-peg, which effectively reorganizes the binding pocket. The AIMD simulations show greater spatial availability for structural reorganization and exchange of water molecules for Eu-pypa-peg complex, relative to Eu-octapa. The observed linearly increasing trend of chelate stabilities across the 4*f* series is similar to that of H_4_edta. The addition of a polyethylene glycol chain averts solubility challenges observed with H_4_octapa. The presence of strongly electron-inducing 2-methylpicolinate moieties is also manifested in the observed differences in chelate stabilities of trivalent lanthanides and actinides. For H_4_octapa, the enhanced actinide/lanthanide differentiation, as inhered from the *β*_101_ ratio for the coordination of Am^3+^ and Nd^3+^, matches that observed for conventional APC reagents. H_4_pypa-peg shows a small improvement in the preference for actinide binding, which may be assigned to the presence of pyridine.

### Supplementary Information

Supplementary Information.

## Data Availability

The datasets used and analyzed during this study are available from the corresponding authors upon reasonable request.
