# Supplementary material for: Tuning aminopolycarboxylate chelators for efficient complexation of trivalent actinides
Source: Sci Rep. 2023 Oct 19;13:17855. doi: 10.1038/s41598-023-44106-6 (PMC10587169; doi:10.1038/s41598-023-44106-6)
Supplement: Supplementary file 1 — Supplementary Information. [file 41598_2023_44106_MOESM1_ESM.pdf]

Supplemental Information for

Tuning aminopolycarboxylate chelators for efficient complexation of trivalent actinides

Corey D. Pilgrim[1,2*], Travis S. Grimes[1], Clayn Smith[1], Colt R. Heathman[1], Jopaul Mathew[3], Santa Jansone-Popova[3], Santanu Roy[3], Debmalya Ray[3], Vyacheslav S. Bryantsev[3], and Peter R. Zalupski[1*]

[1]*Aqueous Separations and Radiochemistry, Idaho National Laboratory, Idaho Falls, Idaho, 83415, United States*

[2]*Glenn T. Seaborg Institute, Idaho National Laboratory, Idaho Falls, Idaho, 83415, United States*

[3]*Chemical Sciences Division, Oak Ridge National Laboratory, Oak Ridge, Tennessee, 37831, United States*

1.) Experimental procedure and characterization data for H_4pypa-peg ii

2.) Spectrophotometric titration solution parameters for H_4octapa and H_4pypa-peg vi

3.) Phase transfer kinetic plots for radiotracer M^{3+} partitioning with H_4octapa and H_4pypa-peg vii

4.) Additional Computational Details viii

5.) Potentiometric curve for the back-titration of H_4octapa using $HClO_4$ xii

6.) Fluorescence lifetime decay measurements and the analyses data for H_4pypa-peg at varying aqueous acidities xiii

7.) Potentiometric curve for the forward- and back-titration of H_4edta xiv

8.) H_4octapa dependencies on the partitioning of Eu^{3+}, Am^{3+} and Cf^{3+} collected at multiple p[H^+] conditions xv

9.) H_4pypa-peg dependencies on the partitioning of Eu^{3+}, Am^{3+} and Cf^{3+} collected at multiple p[H^+] conditions xvii

10.) Verification of stoichiometry of metal extraction by HDEHP and metal complexation by H_4octapa xix

11.) Verification of stoichiometry of metal extraction by HDEHP and metal complexation by H_4pypa-peg xx

12.) Listing of acid dissociation constants and Nd^{3+} and Am^{3+} complexation constants for H_4edta, H_5dtpa and H_6ttha determined in 2.0 M (Na^+/H^+)ClO_4 xxi

13.) Spectrophotometric titration results for Nd^{3+} and Am^{3+} complexation with H_4edta, H_5dtpa and H_6ttha in 2.0 M (Na^+/H^+)ClO_4 xxii

14.) Supplemental Information References xxv

1. Experimental procedure and characterization data for H4pypa-peg

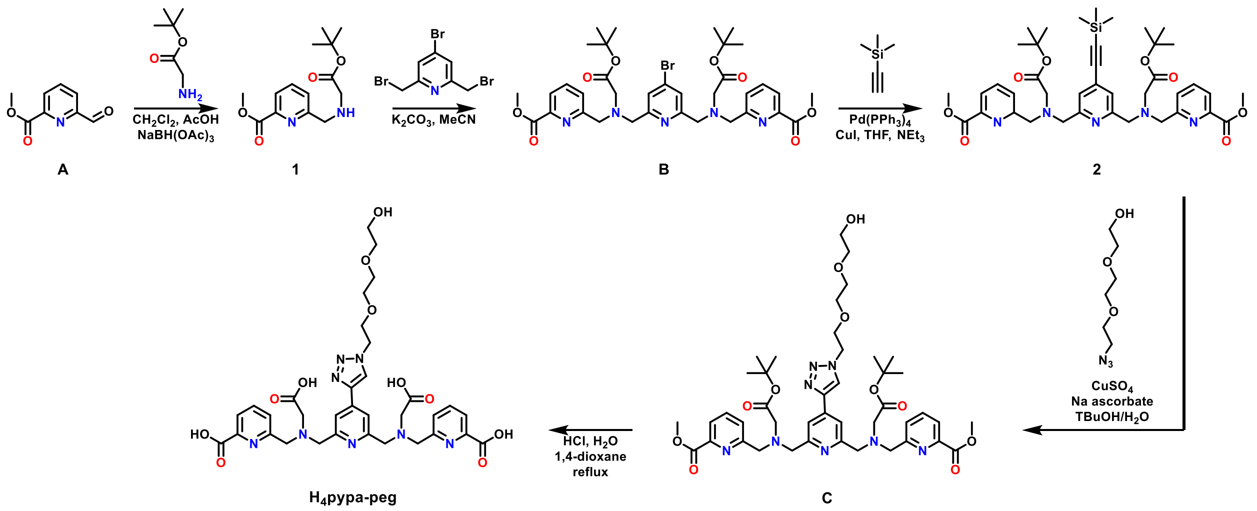

Figure S1. Synthesis of **H4pypa-peg**.

Compound **1** was synthesized according to reported procedure by Abdel-Magid and co-workers.[S1]

Synthesis of compound **B**, dimethyl 6,6'-((((4-bromopyridine-2,6-diyl)bis(methylene))bis((2-(tert-butoxy)-2-oxoethyl)azanediyl))bis(methylene))dipicolinate: a mixture of compound **1**, methyl 6-(((2-(tert-butoxy)-2-oxoethyl)amino)methyl)picolinate, (0.058 mol, 16.3 g), 4-bromo-2,6-bis(bromomethyl)pyridine (0.029 mol, 10.0 g), sodium carbonate (0.29 mol, 30.82 g) in anhydrous acetonitrile (350 mL) was stirred in a 1 L round-bottom flask equipped with stir bar at room temperature for 24 hours. Then, DI water was added to the reaction mixture and product extracted with EtOAc. The organic phase was washed with saturate sodium chloride solution and dried over $MgSO_4$. After filtration, the solvent was removed by evaporation under reduced pressure to afford the crude product, which was purified via column chromatography on silica gel using 70-90% gradient of EtOAc in hexanes to afford compound **B** as an orange oil, 10.7 g, 50% yield. **^{1}H NMR** (400 MHz, $CDCl_3$) δ 8.00 (dd, *J* = 7.39, 1.31 Hz, 2H), 7.87-7.80 (m, 4H), 7.66 (s, 2H), 4.07 (s, 4H), 3.99 (s, 6H), 3.92 (s, 4H), 3.35 (s, 4H), 1.46 (s, 18H).

Synthesis of compound **2**, methyl 6-(((2-(tert-butoxy)-2-oxoethyl)((6-(((2-(tert-butoxy)-2-oxoethyl)((6-(methoxycarbonyl)-2H-1l2-pyridin-2-yl)methyl)amino)methyl)-4-((trimethylsilyl)ethynyl)pyridin-2-yl)methyl)amino)methyl)picolinate: to a solution of **B** (10.7 g, 0.014 mol) in degassed THF:NEt_3 (3:1, 400 mL) was added ethynyltrimethylsilane (3.6 mL, 0.026 mol). The solution was degassed for 5 additional minutes before adding $Pd(PPh_3)_4$ (1.66 g, 10 mol%) and CuI (0.27 g, 10 mol%) under inert atmosphere. The reaction mixture was heated at 50 °C for 12 hours. Afterwards, the reaction mixture was allowed to cool to room temperature and filtered through a short plug of Celite, rinsed with EtOAc. The solvent was removed by evaporation under reduced pressure to afford crude products. The product was purified via column chromatography on silica gel using 80-100% gradient of EtOAc in hexanes to afford **2** as a brown oil, 8.4 g, 77% yield. **^{1}H NMR** (400 MHz, $CDCl_3$) δ 7.99 (d, *J* = 7.55 Hz, 2H), 7.90 (d, *J* = 7.68 Hz, 2H), 7.81 (app t, *J* = 7.71 Hz, 2H), 7.46 (s, 2H), 4.08 (s, 4H), 3.98 (s, 6H), 3.90 (s, 4H), 3.33 (s, 4H), 1.45 (s, 18H), 0.26 (s, 9H).

Synthesis of compound **C**, dimethyl 6,6'-((((4-(1-(2-(2-(2-hydroxyethoxy)ethoxy)ethyl)-1H-1,2,3-triazol-4-yl)pyridine-2,6-diyl)bis(methylene))bis((2-(tert-butoxy)-2-oxoethyl)azanediyl))bis(methylene)) dipicolinate: To a stirred solution of compound **2** (4.3 g, 5.66 mmol), $CuSO_4$ (0.18 g, 1.13 mmol), sodium ascorbate (0.45 g, 2.26 mmol) in *t*BuOH:H_2O (1:1, 180 mL) was added 2-(2-(2-azidoethoxy)ethoxy)ethan-1-ol (0.99 g 0.5 M solution, 5.66 mmol) and K_2CO_3 (0.78 g, 5.66 mmol). The reaction mixture was stirred at room temperature for 12 hours. Afterwards, CH_2Cl_2 was added to the reaction mixture. The organic phase was washed 3x with 5% $_{aq}NH_4OH$ solution, followed by saturated sodium chloride solution. The organic layer was dried over $MgSO_4$ and the solvent was removed by evaporation under reduced pressure affording the crude product. The product was purified via column chromatography on neutral alumina using 0-30% gradient of MeOH in CH_2Cl_2 to afford compound **C** as a colorless oil, 4.8 g, 95% yield.

Synthesis of **H_4pypa-peg**, 6,6'-((((4-(1-(2-(2-(2-hydroxyethoxy)ethoxy)ethyl)-1H-1,2,3-triazol-4-yl)pyridine-2,6-diyl)bis(methylene))bis((carboxymethyl)azanediyl))bis(methylene))dipicolinic acid: a mixture of compound **C** (64.8 g, 5.65 mmol), 1,4-dioxane (37 mL), DI H_2O (2.2 mL), and concentrated hydrochloric acid (12 N, 6.0 mL) was refluxed for 12 hours under inert atmosphere. Afterwards, the solvent was evaporated under reduced pressure affording crude product. This crude product was loaded onto a celite precolumn and purified via column chromatography on reverse-phase C18 AQ column, eluting with 100% water for 5 minutes, then 0-50% MeOH in water gradient over 30 minutes. The final product was obtained as a pale-yellow foam, 1.66 g, 41% yield. **^{1}H NMR** (400 MHz, DMSO-d_6) δ 12.53 (br s, 2H), 8.73 (s, 1H), 7.94 – 7.87 (m, 4H), 7.80 (s, 2H), 7.76 (d, J = 7.65 Hz, 2H), 4.62 (app t, J = 4.85 Hz, 2H), 4.03 (s, 4H), 3.98 (s, 4H), 3.89 (app t, J = 4.96 Hz, 2H), 3.57 – 3.55 (m, 2H), 3.50 – 3.42 (m, 8H), 3.36 (app t, J = 4.81 Hz, 2H). **^{13}C NMR** (126 MHz, DMSO-d_6) δ 173.3, 166.2, 159.5, 159.1, 147.6, 144.2, 139.0, 137.8, 125.9, 123.7, 123.1, 116.9, 72.3, 69.6 (2 accidental equivalent carbons), 68.6, 60.2, 59.1, 58.9, 54.5, 49.8. **EA** for $C_{33}H_{41}Cl_3N_8O_{11}$: C, 54.84; H, 5.30 N, 15.50; found: C, 52.90; H, 5.48; N, 14.78.

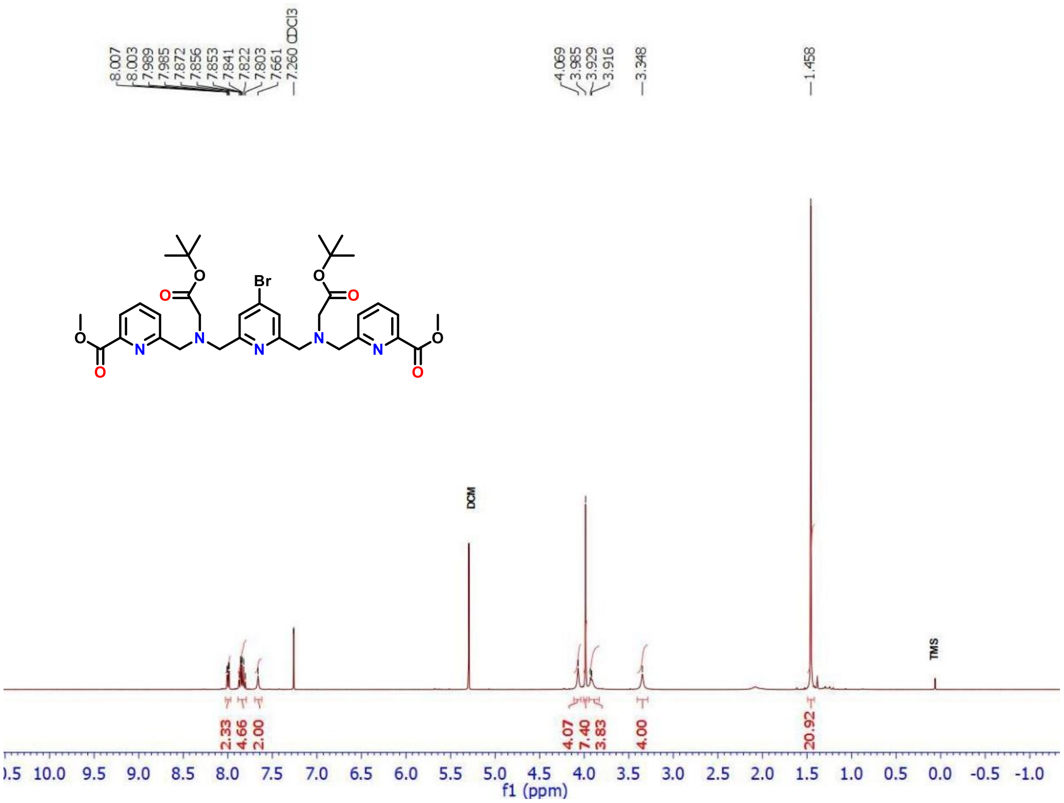

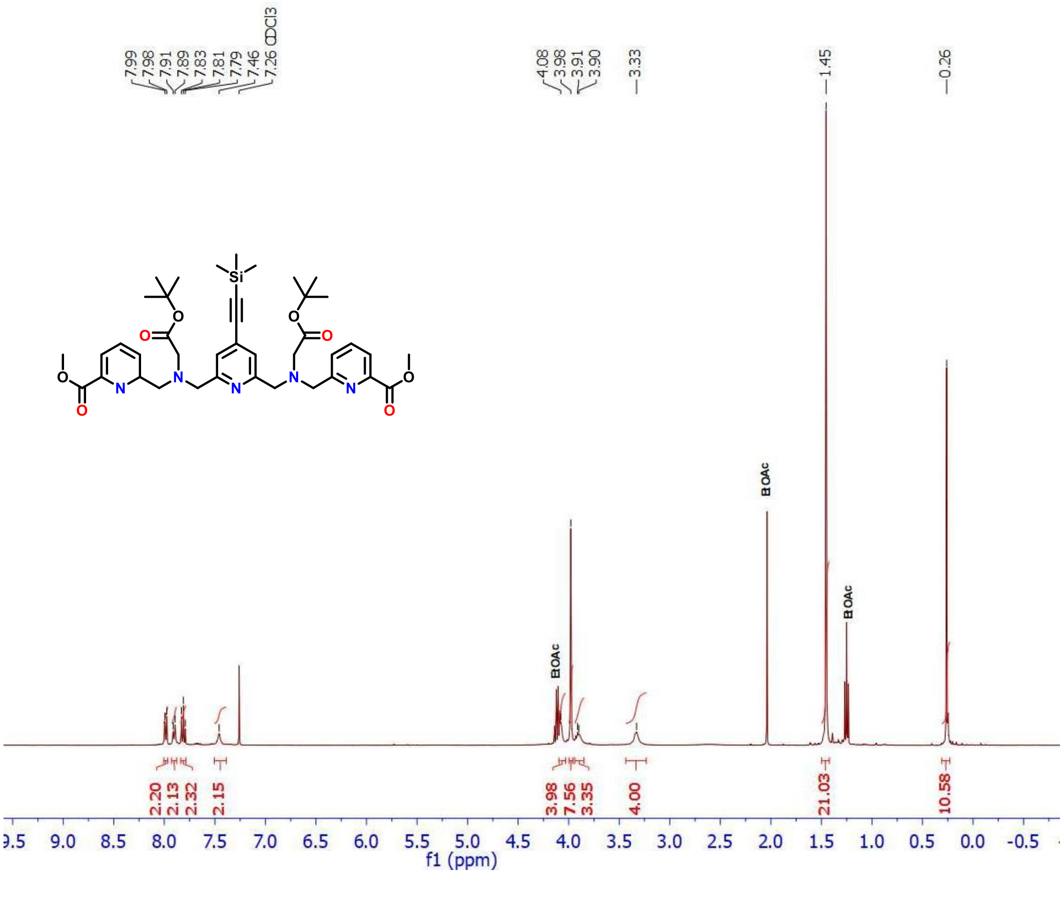

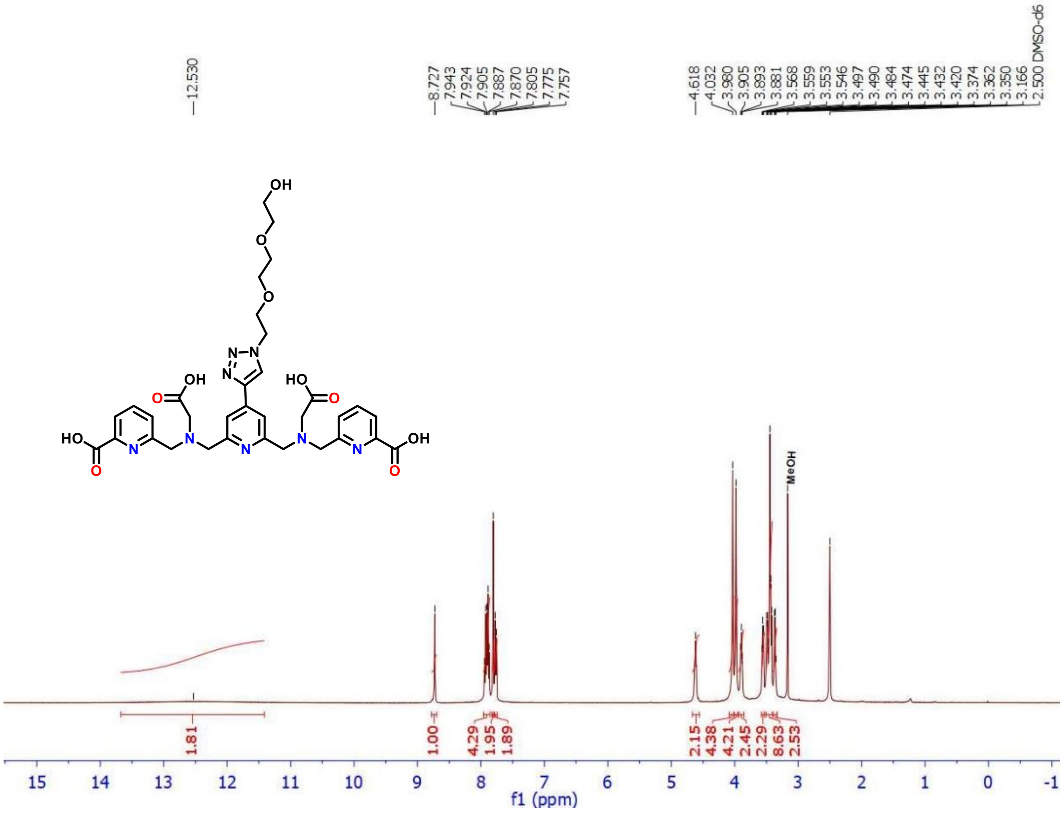

12.530
8.727
7.943
7.924
7.905
7.887
7.870
7.805
7.775
7.757
4.618
4.032
3.980
3.905
3.893
3.881
3.568
3.559
3.553
3.546
3.497
3.490
3.484
3.474
3.445
3.432
3.420
3.374
3.362
3.350
3.166
2.500 DMSO-d6
MeOH
1.81
1.00
4.29
1.95
1.89
2.15
4.38
4.21
2.45
2.29
8.63
2.53
f1 (ppm)

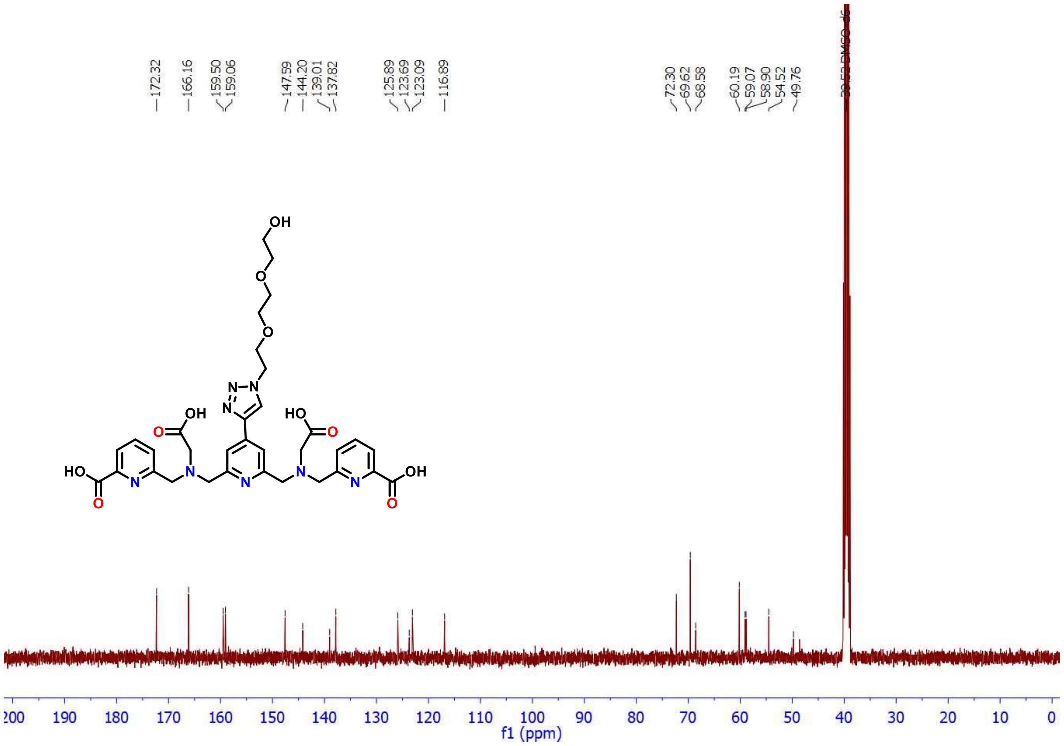

172.32
166.16
159.50
159.06
147.59
144.20
139.01
137.82
125.89
123.69
123.09
116.89
72.30
69.62
68.58
60.19
59.07
58.90
54.52
49.76
f1 (ppm)

2. Spectrophotometric titration parameters

H_4octapa

Nd^{3+}: The titrand solution consisting of 1.980 mL of 7.57 mM $Nd(ClO_4)_3$ at $p[H^+] = 1.61$ was blended with 1.965 mL of titrant solution containing 7.62 mM Nd^{3+}, 18.57 mM H_4octapa, at $p[H^+] \approx 5.0$-6.0.

Am^{3+}: The titrand solution consisting of 0.842 mL of 0.810 mM $Am(ClO_4)_3$ at $p[H^+] =1.66$ was blended with 0.811 mL of titrant solution containing 0.815 mM Am^{3+}, 8.95 mM H_4octapa, at $p[H^+] \approx 5.0 – 6.0$.

H_4pypa-peg

Nd^{3+}: The titrand solution consisting of 0.801 mL of 9.82 mM $Nd(ClO_4)_3$ at $p[H^+] = 1.61$ was blended with 1.176 mL of titrant solution containing 9.91 mM Nd^{3+} and 24.58 mM H_4pypa-peg in with $p[H^+] \approx 3.0 – 4.0$.

Am^{3+}: The titrand solution consisting of 0.806 mL of 0.791 mM $Am(ClO_4)_3$ at $p[H^+] = 1.41$ was blended with 0.787 mL of titrant solution containing 0.801 mM Am^{3+} and 15.09 mM H_4pypa-peg at $p[H^+] \approx 5 – 6$.

Cm^{3+}: The titrand solution consisting of 0.809 mL of 1.00 mM $Cm(ClO_4)_3$ at $p[H^+] = 1.34$ was blended with 0.283 mL of titrant solution containing 1.00 mM Cm^{3+} and 15.10 mM H_4pypa-peg at $p[H^+] \approx 5 – 6$.

3. Phase transfer kinetic plots for radiotracer M^{3+} partitioning with H_4octapa and H_4pypa-peg

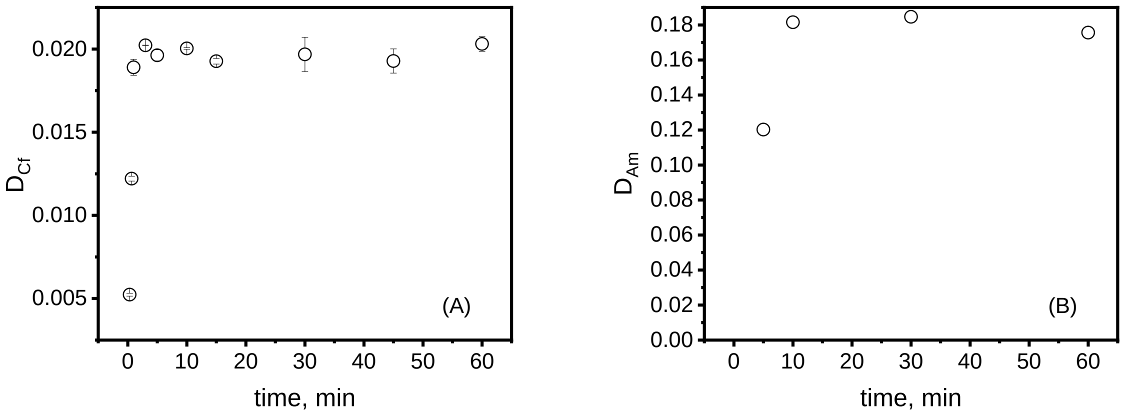

Figure S2. Time dependencies for the liquid-liquid partitioning of (A) Cf $^{3+}$ and (B) Am^{3+} between organic phase containing HDEHP in octane and aqueous phase containing H_4octapa. (A) [HDEHP] = 0.014 M, [H_4octapa] = 0.5 mM, p[H^+] = 2.0. (B) [HDEHP] = 0.10 M, [H_4octapa] = 0.1 mM, p[H^+] = 2.0.

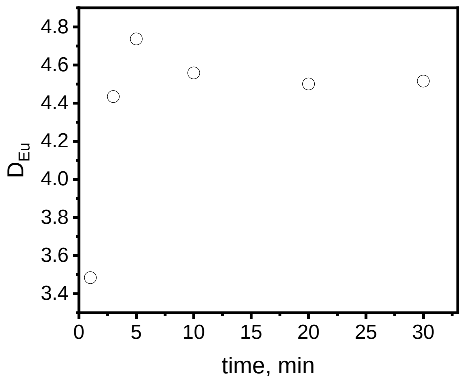

Figure S3. Time dependencies for the liquid-liquid partitioning of (A) Eu^{3+} between organic phase containing HDEHP in *n*-octane and aqueous phase containing H_4pypa-peg. Org: [HDEHP] = 0.014 M in *n*-octane, Aq: [H_4pypa-peg] = 0.3 mM, p[H^+] = 2.0.

4. Additional Computational Details.

The crystal structures of octapa complexes with La[S2] and Gd,[S3] $[Ln(octapa)(H_2O)_2]^-$, as well as the initial geometries obtained from the Eu-EDTA complex by replacing carboxylate with picolinate groups were used as starting geometries for the $[Eu(octapa)(H_2O)_2]^-$ complex. Next, geometry optimization was performed at the B3LYP/Eu-LC(7s6p5d)/[5s4p3d2f])/6-31+G(d) level of theory[S4,S5] using Gaussian 16[S6] software to sample possible coordination modes and identify the low energy structures in implicit solvent. The most stable geometry of $[Eu(octapa)(H_2O)_2]^-$ having C_2 symmetry was highly similar to two crystal structures.[S2,S3]

For pypa-peg ligand we did not have any experimental crystal structure. Thus, we searched the Cambridge Structural Database (CSD) for similar crystal structure motifs. Three such structures were identified (CSD Refcodes LUZWEL[S7], QEYDAC[S8] and IWOXAW[S9]) and edited to make the three different starting geometries for $[Eu(pypa\text{-}peg)]^-$. To expedite the computations, we replaced the long alkyl chain attached to the five-membered ring of the pypa-peg ligand with the H atom. After initial geometry optimization at the B3LYP/Eu-LC(7s6p5d)/[5s4p3d2f])/6-31+G(d) level of theory[S4,S5] using Gaussian 16[S6] software and implicit solvent model for water, we added one explicit H_2O molecule to each structure and reoptimized at the same level of theory. We found that the initial geometries obtained from LUZWEL and IWOXAW were more stable compared to the one obtained from the QEYDAC structure. Further, we took those two geometries and performed a conformational search using a systematic rotor search method in Avogadro.[S10] Next, we took top 20 candidates (out of 728) from each conformation search and reoptimized them at the B3LYP/Eu-LC(7s6p5d)/[5s4p3d2f])/6-31+G(d) level of theory[S4,S5] using Gaussian 16[S6] software and implicit solvent model. During the conformational search in Avogadro the metal-O and metal-N bond lengths were constrained to 2.45 Å and 2.75 Å, respectively. This approach was taken in order to guarantee the generation of ten-coordinate initial geometries. Geometry optimization of 40 initial geometries obtained from the initial conformation search gave two types of geometries. In the first case, the explicit water molecule was bounded to the Eu(III) center (in this case one of the N atoms of pypa had

slightly elongated bond length) with the coordination number (CN) 10 and in the second case, the explicit water molecule was bridging between two carboxylate groups (in this case the H_2O molecule was not directly attached to the Eu(III) center) and has the coordination number 9. Since experimentally the H_2O molecule is attached to the metal center, we further proceeded with the most stable CN = 10 structure. In order to keep the periodic box size smaller, we removed the 5-membered triazole ring for molecular dynamics simulations.

In addition to AIMD simulations performed using VASP, we carried out two independent AIMD simulations for $[Eu(pypa)(H_2O)_n]^-$ (n=0,1) complexes using the CP2K software package.[S11] For $[Eu(pypa)(H_2O)]^-$ complex we used the same initial geometry as for the VASP AIMD run, whereas for $[Eu(pypa)]^-$ we took the geometry snapshot from the last frame of the VASP AIMD simulation. AIMD simulations were performed at 298.15 K using the Nosé-Hover thermostat with a timestep of 1.0 fs. The -1 charge on the complex was compensated by a uniform background charge. Norm conserving pseudopotentials were used for core electrons[S12] and the DZVP quality Gaussian type basis sets were used for all atoms. An auxiliary plane-wave basis set[S13] with 600 Ry cutoff criteria was used for calculating electrostatic terms. All AIMD calculations were done with the Γ point approximation. The PBE GGA functional[S14,S15] was used to describe the exchange-correlation energy together with the DFT-D3 method of Grimme[S16] for dispersion interactions. For $[Eu(pypa)(H_2O)]^-$ we obtained a trajectory of ~80 ps whereas for $[Eu(pypa)]^-$ we obtained a trajectory of ~15 ps. Potential energies for the last 10,000 steps (**Figure S6**) were used to assess the relative stability of the $[Eu(pypa)(H_2O)]^-$ and $[Eu(pypa)]^-$ complexes.

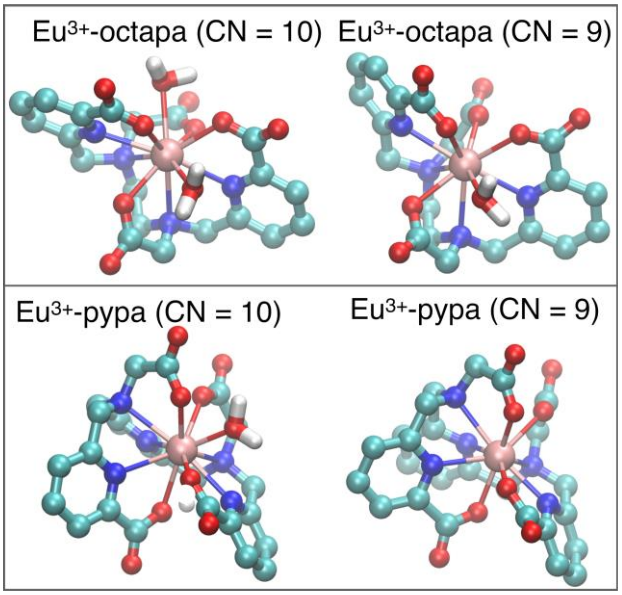

Figure S4. Snapshots from AIMD for different Eu^{3+}-octapa and Eu^{3+}-pypa coordination states. Hydrogen atoms on the ligands are not shown for clarity.

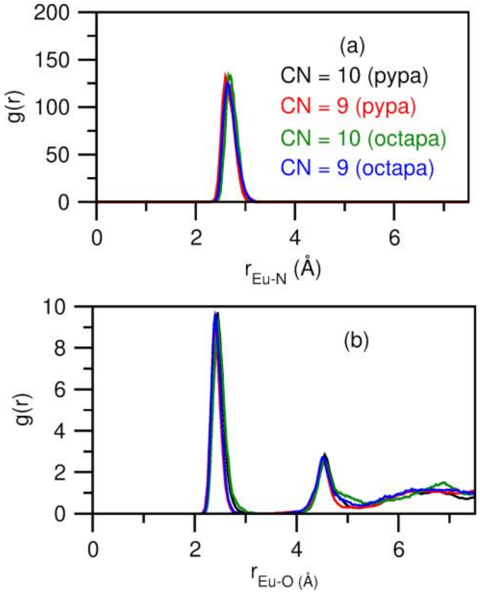

Figure S5. The Eu-N (a) and Eu-O (b) radial distribution functions (RDF) for Eu^{3+}-octapa and Eu^{3+}-pypa complexes in different coordination states.

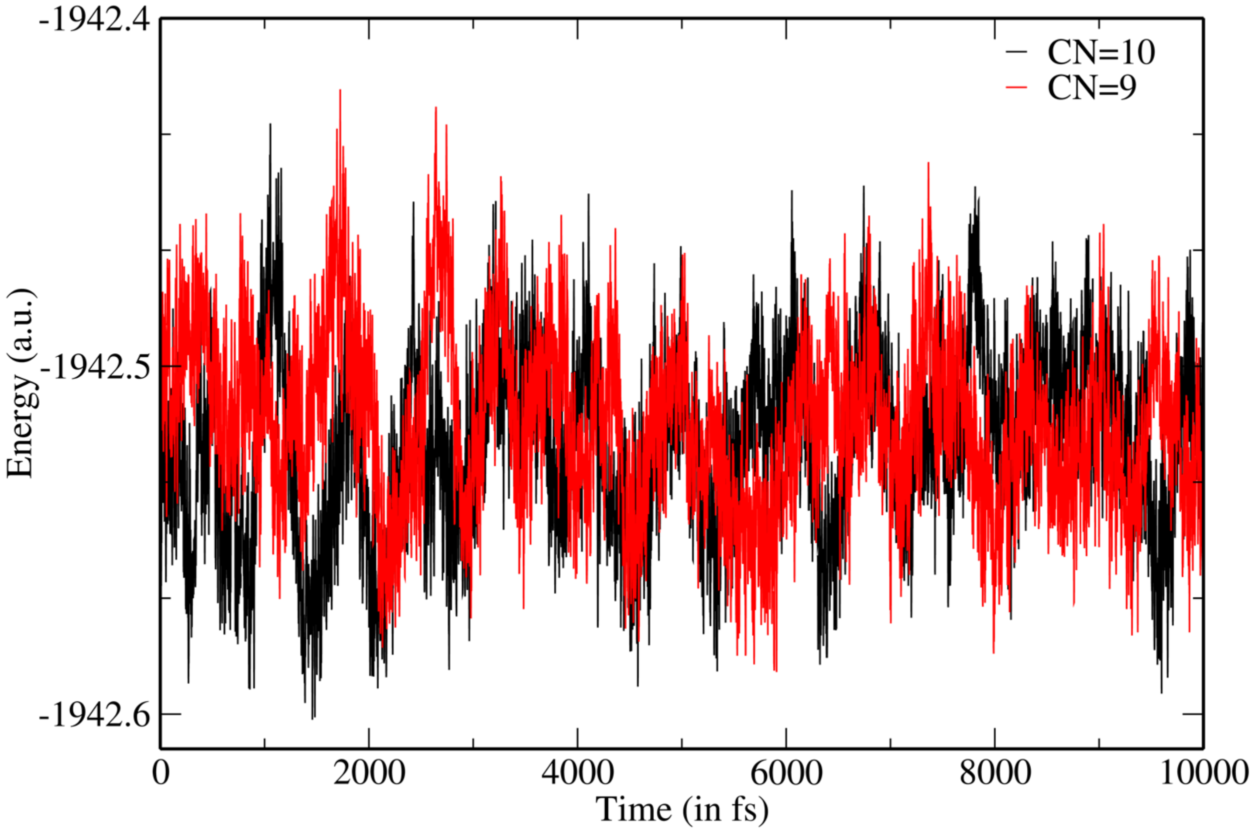

Figure S6. The overlap of the potential energies for $[Eu(pypa)(H_2O)_n]^-$ (n=0,1) complexes from the last 10 ps of AIMD simulations at 298.15 K using the CP2K software package.

Table S1. Bond distances (Å) in the Eu^{3+}-pypa complex obtained from the AIMD simulation at 298.15 K using the CP2K software package.

	$[Eu(pypa)(H_2O)_n]^-$
	n = 1
Eu-$O_{\mathbf{1\text{-}pyr}}$	2.46 ± 0.10
Eu-$O_{\mathbf{2\text{-}acet}}$	2.52 ± 0.10
Eu-$O_{\mathbf{3\text{-}acet}}$	2.46 ± 0.10
Eu-$O_{\mathbf{4\text{-}pyr}}$	2.48 ± 0.10
Eu-$O_{\mathbf{W1}}$	2.59 ± 0.13
Eu-$N_{\mathbf{1\text{-}pyr}}$	2.69 ± 0.10
Eu-$N_{\mathbf{2\text{-}amine}}$	2.87 ± 0.11
Eu-$N_{\mathbf{3\text{-}amine}}$	2.69 ± 0.10
Eu-$N_{\mathbf{4\text{-}pyr}}$	2.62 ± 0.10
Eu-$N_{\mathbf{5\text{-}pyr}}$	2.63 ± 0.10

5. Potentiometric curve for the back-titration of H_4octapa using $HClO_4$.

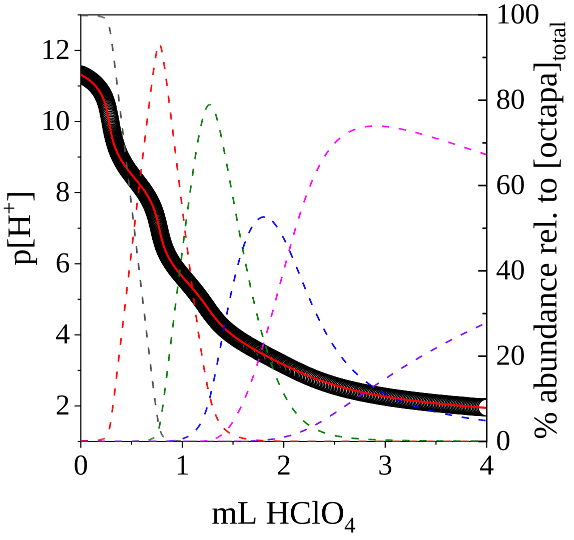

Figure S7. Potentiometric p[H$^+$] trend collected for H_4octapa protonation titration at $T = 20.0 \pm 0.1$°C and $I = 2.00 \pm 0.01$ M (Na^+/H^+)ClO_4. **(A)** Titrand: $V_{init} = 10.383$ mL, $C_{H4octapa} = 4.759$ mM, $C_{OH-} = 2.690$ mM. Titrant: 0.100 M $HClO_4$ and 1.900 M $NaClO_4$. (○) Experimental p[H$^+$], (——) calculated p[H$^+$], (---) L^{4-}, (---) HL^{3-}, (---) H_2L^{2-}, (---) H_3L^-, (---) $H_4L_{(Aq)}$, (---) H_5L^+.

6. Potentiometric curve for the forward- and back-titration of H_4edta.

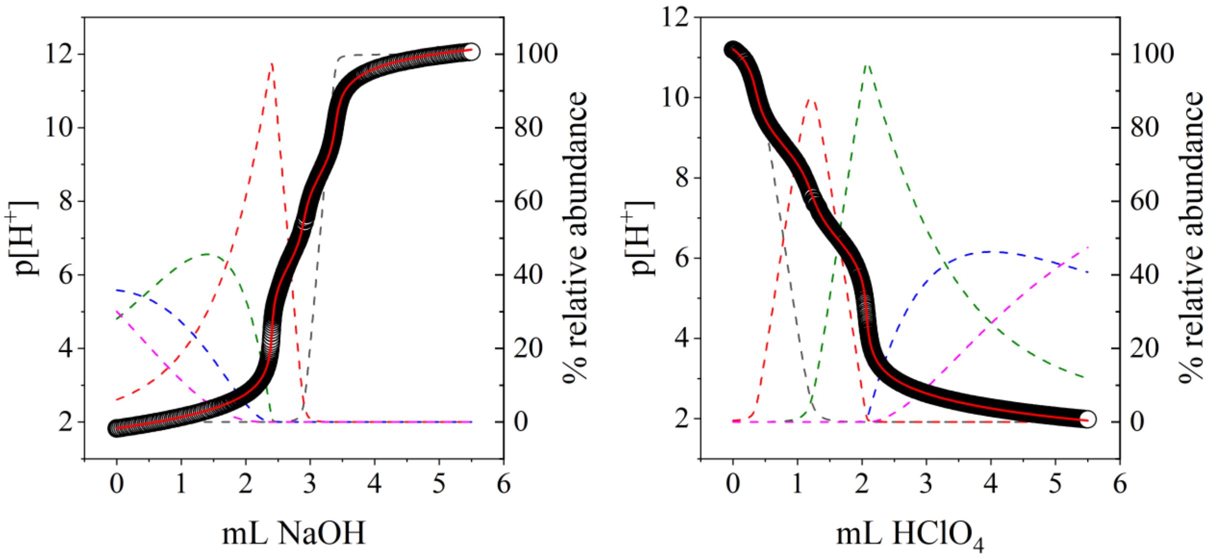

Figure S8. Potentiometric titration curves for H_4edta.

7. Fluorescence lifetime decay measurements and the analyses data for H_4pypa-peg at varying aqueous acidities.

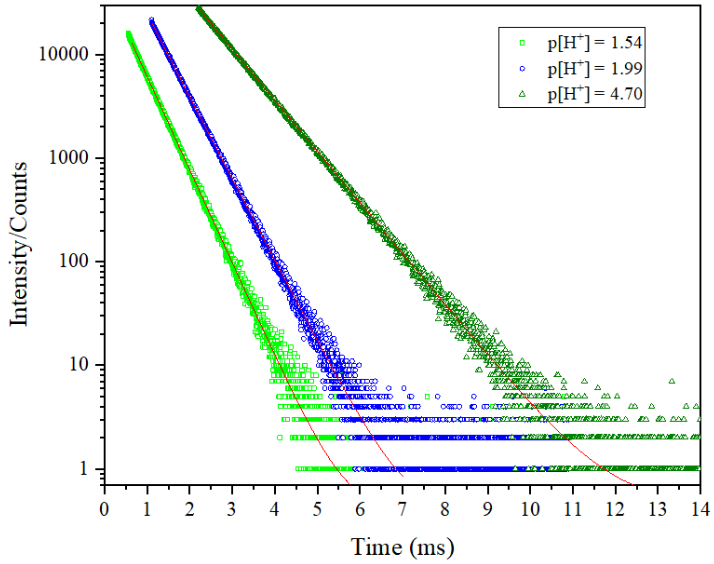

Figure S9: Fluorescence lifetime decay patterns for Eu^{3+} in presence of H_4pypa-peg at p[H^+] = 1.54, 1.99, and 4.70. I = 2.00 M (H^+,Na^+)ClO_4. Red lines indicate the monoexponential (p[H^+] = 1.54, 4.70) and biexponential (p[H^+] = 1.99) fits of the data.

Table S2. Luminescence lifetime decay (τ), calculated hydration number (η_{H2O}), and calculated species distribution for Eu^{3+} and H_4pypa-peg mixtures.

					**Abundance of species, relative to total Eu^{3+}**		
**p[H^+]**	τ_1 (μs)	η_{H2O}(± 0.5)	τ_2 (μs)	η_{H2O}(± 0.5)	Free Eu^{3+}	Eu(Hpypa-peg)$_{(aq)}$	[Eu(pypa-peg)]$^-$
1.54	482.9	1.7	—	—	5.9%	68.2%	25.9%
1.99*	401.5	2.2	557.9	1.4	—	48.3%	51.7%
4.70	879.7	0.8	—	—	—	0.4%	99.8%

*Required a biexponential fit of the luminescent decay data.

Equation used to calculate the waters of hydration: $\eta_{\mathrm{H2O}} = 1.05\ x\ 10^{-3} k_{obs}(Eu) - 0.44$

8. H_4octapa dependencies on the partitioning of Eu^{3+}, Am^{3+} and Cf^{3+} collected at multiple p[H^+] conditions.

Table S3. Metal distribution ratios collected for the H_4octapa dependencies on the partitioning of Cf^{3+}. Organic phase contained 0.014 M HDEHP in octane. I = 2.00 M (H^+,Na^+)ClO_4. T = 20.0 ± 0.5 °C.

p[H^+] 1.80		p[H^+] 1.89		p[H^+] 2.01	
[octapa], mM	D_{Cf}	[octapa], mM	D_{Cf}	[octapa], mM	D_{Cf}
0.000	37 ± 2	0.000	63 ± 3	0.000	181.4 ± 0.3
0.010	0.782 ± 0.003	0.010	0.62 ± 0.03	0.010	0.401 ± 0.002
0.020	0.38 ± 0.01	0.020	0.275 ± 0.002	0.020	0.200 ± 0.003
0.050	0.141 ± 0.002	0.049	0.110 ± 0.002	0.049	0.074 ± 0.001
0.099	0.066 ± 0.004	0.100	0.058 ± 0.001	0.099	0.037 ± 0.002
0.151	0.043 ± 0.003	0.150	0.036 ± 0.001	0.149	0.024 ± 0.001
0.200	0.036 ± 0.004	0.202	0.025 ± 0.002	0.200	0.018 ± 0.001
0.302	0.023 ± 0.001	0.302	0.018 ± 0.001	0.299	0.012 ± 0.001

Table S4. Metal distribution ratios collected for the H_4octapa dependencies on the partitioning of Eu^{3+}. Organic phase contained 0.014 M HDEHP in octane. I = 2.00 M (H^+,Na^+)ClO_4. T = 20.0 ± 0.5 °C.

p[H^+] 1.78		p[H^+] 1.88		p[H^+] 1.99	
[octapa], mM	D_{Eu}	[octapa], mM	D_{Eu}	[octapa], mM	D_{Eu}
0.000	18.4 ± 0.8	0.000	34.3 ± 0.4	0.000	62.6 ± 0.3
0.010	10.8 ± 0.6	0.010	10.9 ± 0.4	0.010	10.8 ± 0.6
0.020	7.0 ± 0.3	0.020	5.78 ± 0.01	0.020	5.19 ± 0.08
0.050	3.38 ± 0.07	0.049	2.94 ± 0.07	0.049	3.0 ± 0.3
0.099	1.78 ± 0.04	0.100	1.47 ± 0.02	0.099	1.116 ± 0.005
0.151	1.24 ± 0.01	0.150	0.91 ± 0.01	0.149	0.721 ± 0.007
0.200	0.972 ± 0.001	0.202	0.675 ± 0.008	0.200	0.56 ± 0.02
0.302	0.67 ± 0.02	0.302	0.481 ± 0.004	0.299	0.362 ± 0.004

Table S5. Metal distribution ratios collected for the H_4octapa dependencies on the partitioning of Am^{3+}. Organic phase contained 0.10 M HDEHP in octane. I = 2.00 M (H^+,Na^+)ClO_4. T = 20.0 ± 0.5 °C.

**p[H$^+$] 1.80**		**p[H$^+$] 1.88**		**p[H$^+$] 2.02**	
[octapa], mM	D_{Am}	[octapa], mM	D_{Am}	[octapa], mM	D_{Am}
0.000	35 ± 3	0.000	52 ± 7	0.000	109 ± 4
0.010	2.4 ± 0.1	0.010	1.92 ± 0.01	0.010	1.64 ± 0.02
0.020	1.21 ± 0.02	0.020	0.969 ± 0.003	0.020	0.731 ± 0.002
0.050	0.460 ± 0.003	0.049	0.379 ± 0.003	0.049	0.392 ± 0.009
0.099	0.231 ± 0.001	0.100	0.211 ± 0.008	0.099	0.174 ± 0.004
0.151	0.169 ± 0.002	0.150	0.130 ± 0.003	0.149	0.105 ± 0.008
0.200	0.120 ± 0.001	0.202	0.096 ± 0.001	0.200	0.067 ± 0.002
0.302	0.082 ± 0.001	0.302	0.066 ± 0.001	0.299	0.045 ± 0.001

9. H_4pypa-peg dependencies on the partitioning of Eu^{3+}, Am^{3+} and Cf^{3+} collected at multiple p[H^+] conditions.

Table S6. Metal distribution ratios collected for the H_4pypa-peg dependencies on the partitioning of Eu^{3+}. Organic phase contained 0.014 M HDEHP in octane. I = 2.0 M (H^+/Na^+)ClO_4. T = 20.0 ± 0.5 °C.

**p[H^+] 2.00**		**p[H^+] 2.09**		**p[H^+] 2.25**	
[pypa-peg], mM	D_{Eu}	[pypa-peg], mM	D_{Eu}	[pypa-peg], mM	D_{Eu}
0.000	60.691 ± 0.003	0.000	110 ± 4	0.000	314 ± 28
0.050	9.9 ± 0.3	0.050	7.42 ± 0.05	0.049	5.1 ± 0.1
0.099	5.0 ± 0.2	0.100	3.67 ± 0.07	0.100	2.2 ± 0.4
0.149	3.5 ± 0.1	0.150	2.48 ± 0.02	0.151	1.4 ± 0.1
0.199	2.58 ± 0.02	0.201	1.7 ± 0.1	0.203	1.01 ± 0.02
0.301	1.75 ± 0.06	0.302	1.19 ± 0.01	0.301	0.67 ± 0.03
0.399	1.29 ± 0.04	0.402	0.95 ± 0.03	0.402	0.52 ± 0.02
0.500	1.03 ± 0.05	0.502	0.72 ± 0.02	0.502	0.402 ± 0.007

Table S7. Metal distribution ratios collected for the H_4pypa-peg dependencies on the partitioning of Am^{3+}. Organic phase contained 0.08 M HDEHP in octane. I = 2.0 M (H^+/Na^+)ClO_4. T = 20.0 ± 0.5 °C.

**p[H^+] 1.79**		**p[H^+] 1.90**		**p[H^+] 2.00**	
[pypa-peg], mM	D_{Am}	[pypa-peg], mM	D_{Am}	[pypa-peg], mM	D_{Am}
0.000	20 ± 2	0.000	39 ± 2	0.000	81.3 ± 0.7
0.151	0.789 ± 0.007	0.049	1.72 ± 0.04	0.050	1.20 ± 0.05
0.203	0.587 ± 0.002	0.100	0.847 ± 0.003	0.100	0.62 ± 0.04
0.301	0.40 ± 0.01	0.150	0.561 ± 0.008	0.150	0.41 ± 0.01
0.402	0.289 ± 0.001	0.201	0.408 ± 0.008	0.201	0.29 ± 0.02
0.502	0.241	0.301	0.279 ± 0.008	0.302	0.195 ± 0.006
		0.401	0.202 ± 0.002	0.402	0.148 ± 0.001
		0.501	0.156 ± 0.004	0.502	0.119 ± 0.001

Table S8. Metal distribution ratios collected for the H_4pypa-peg dependencies on the partitioning of Cf^{3+}. Organic phase contained 0.014 M HDEHP in octane. I = 2.0 M $(H^+/Na^+)ClO_4$. T = 20.0 ± 0.5 °C.

p[H⁺] 1.78		p[H⁺] 1.88		p[H⁺] 1.98	
[pypa-peg], mM	D_{Cf}	[pypa-peg], mM	D_{Cf}	[pypa-peg], mM	D_{Cf}
0.000	29.5 ± 0.4	0.000	46 ± 4	0.000	95.7 ± 0.2
0.010	0.71 ± 0.03	0.010	0.46 ± 0.01	0.010	0.35 ± 0.02
0.020	0.36 ± 0.01	0.020	0.228 ± 0.006	0.020	0.155 ± 0.008
0.049	0.137 ± 0.002	0.049	0.09 ± 0.01	0.050	0.060 ± 0.002
0.100	0.067 ± 0.001	0.100	0.044 ± 0.001	0.100	0.029 ± 0.001
0.151	0.045 ± 0.001	0.150	0.028 ± 0.001	0.150	0.020 ± 0.002
0.203	0.034 ± 0.001	0.201	0.022 ± 0.001	0.201	0.011 ± 0.001
0.301	0.023 ± 0.001	0.301	0.015 ± 0.001	0.302	0.008 ± 0.001
0.402	0.017 ± 0.001	0.401	0.010 ± 0.001	0.402	0.006 ± 0.001
0.502	0.014 ± 0.002	0.501	0.009 ± 0.001	0.502	0.005 ± 0.001

10. Verification of stoichiometry of metal extraction by HDEHP and metal complexation by H_4octapa.

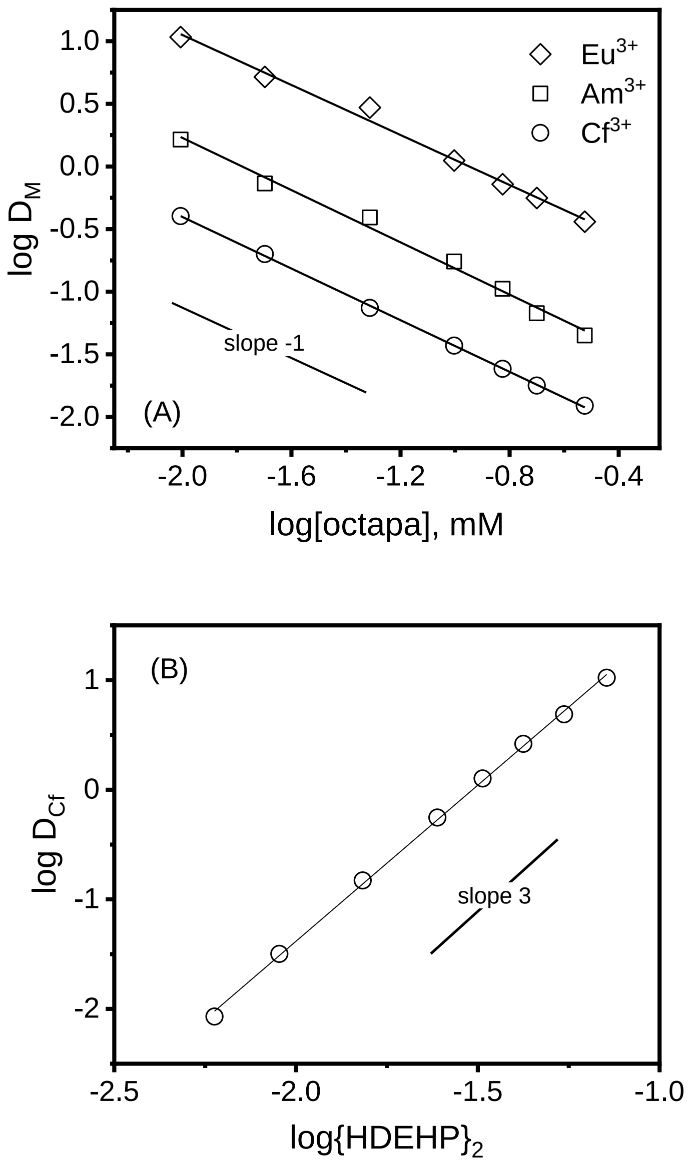

Figure S10. (A) Liquid-liquid partitioning of Cf^{3+}, Am^{3+} and Eu^{3+} between organic phase containing HDEHP in octane and aqueous phase containing varying concentrations of H_4octapa. [HDEHP] = 0.014 M in octane for Cf^{3+} and Eu^{3+}. [HDEHP] = 0.10 M in octane for Am^{3+}. [H_4octapa] = 0.01 - 0.3 mM, p[H^+] = 2.0. (B) Dependence of Cf^{3+} distribution on the activity of HDEHP dimer in octane as calculated using activity coefficient reported by Danesi and Vandegrift.[S2] Org: 0.014 – 0.301 M HDEHP in octane. Aq: 0.5 mM H_4octapa, p[H^+] 2.01, 2.00 M (H^+,Na^+)ClO_4.

11. Verification of stoichiometry of metal extraction by HDEHP and metal complexation by H_4pypa-peg.

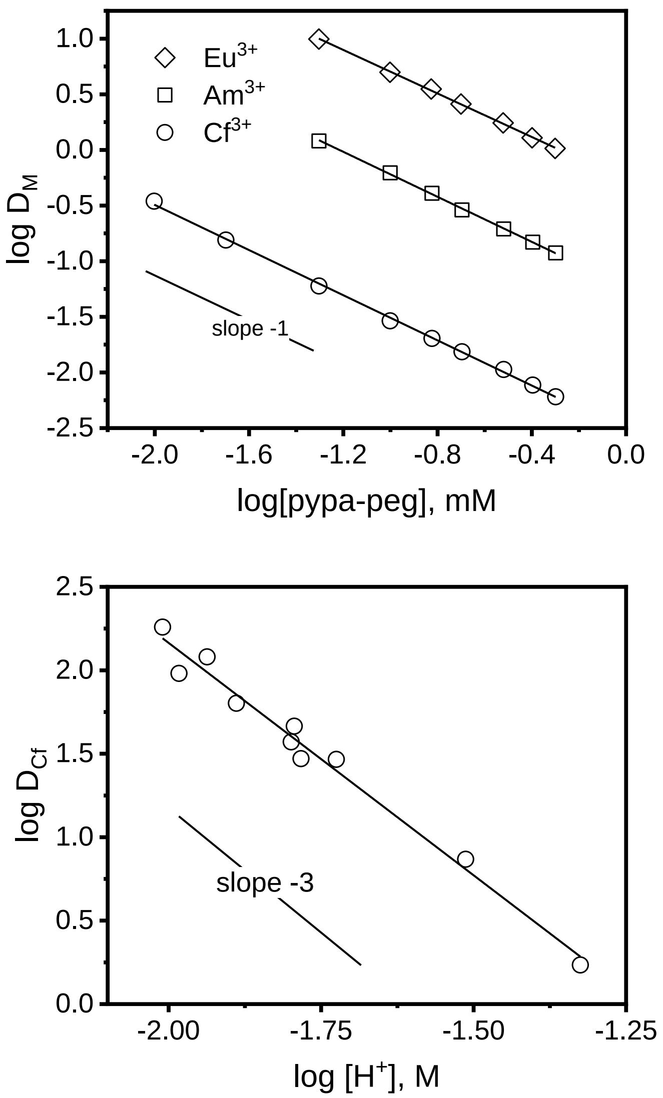

Figure S11. (A) Liquid-liquid partitioning of Eu^{3+}, Am^{3+}, and Cf^{3+} between organic phase containing HDEHP in *n*-octane and aqueous phase containing varying concentrations of H_4pypa-peg. [HDEHP] = 0.014 M in *n*-octane for Eu^{3+} and Cf^{3+}. [HDEHP] = 0.08 M in *n*-octane for Am^{3+}. [H_4pypa-peg] = 0.05 - 0.5 mM for Eu^{3+} and Am^{3+}, p[H^+] = 2.00; [H_4pypa-peg] = 0.01 - 0.5 mM for Cf^{3+}, p[H^+] = 1.98. **(B)** Dependence of Cf^{3+} distribution on the aqueous concentration of hydrogen ion. Org: 0.014 M HDEHP in octane. Aq: 2.00 M (H^+/Na^+)ClO_4, p[H^+] 1.33 - 2.01.

12. Listing of acid dissociation constants and Nd^{3+} and Am^{3+} complexation constants for H_4edta, H_5dtpa and H_6ttha determined in 2.0 M $(Na^+/H^+)ClO_4$.

Table S9. Acid dissociation constants determined for H_4edta, H_5dtpa and H_6ttha determined in I = 2.0 M $(Na^+/H^+)ClO_4$ at 20.0°C.

H_4edta	*n*	pK_n	H_5dtpa	*n*	pK_n	H_6ttha	*n*	pK_n
HL^{3-}	6	8.81(1)	HL^{4-}	8	9.50(1)	HL^{5-}	10	9.48(1)
H_2L^{2-}	5	6.45(1)	H_2L^{3-}	7	8.31(1)	H_2L^{4-}	9	8.67(1)
H_3L^-	4	2.49(1)	H_3L^{2-}	6	5.57(1)	H_3L^{3-}	8	6.27(1)
$H_4L_{(aq)}$	3	2.02(3)	H_4L^-	5	4.38(1)	H_4L^{2-}	7	4.12(1)
H_5L^+	2		$H_5L_{(aq)}$	4	2.53(3)	H_5L^-	6	2.70(1)
H_6L^{2+}	1		H_6L^+	3	2.41(1)	$H_6L_{(aq)}$	5	2.19(4)
			H_7L^{2+}	2		H_7L^+	4	1.86(4)
			H_8L^{3+}	1		H_8L^{2+}	3	
						H_9L^{3+}	2	
						$H_{10}L^{4+}$	1	

Table S10. Conditional stability constants for the complexation of Nd^{3+} and Am^{3+} with H_4edta, H_5dtpa and H_6ttha determined in I = 2.0 M $(Na^+/H^+)ClO_4$ at 20.0°C.

H_4edta	*mhl*	logβ_{mhl}	H_5dtpa	*mhl*	logβ_{mhl}	H_6ttha	*mhl*	logβ_{mhl}
NdL^-	101	14.66(1)	NdL^{2-}	101	20.23(2)	NdL^{3-}	101	21.90(7)
AmL^-	101	TBD(1)	$NdHL^-$	111	21.59(2)	$NdHL^{2-}$	111	25.56(7)
			AmL^{2-}	101	21.45(1)	NdH_2L^-	121	27.7(1)
			$AmHL^-$	111	22.62(2)	NdL^{3-}	101	21.78(2)$^+$
						$NdHL^{2-}$	111	25.42(2)$^+$
						NdH_2L^-	121	27.74(3)$^+$
						AmL^{3-}	101	23.25(3)
						$AmHL^{2-}$	111	27.02(3)
						AmH_2L^-	121	28.84(2)

$^+$determined using potentiometry

13. Spectrophotometric titration results for Nd^{3+} and Am^{3+} complexation with H_4edta, H_5dtpa and H_6ttha in 2.0 M $(Na^+/H^+)ClO_4$.

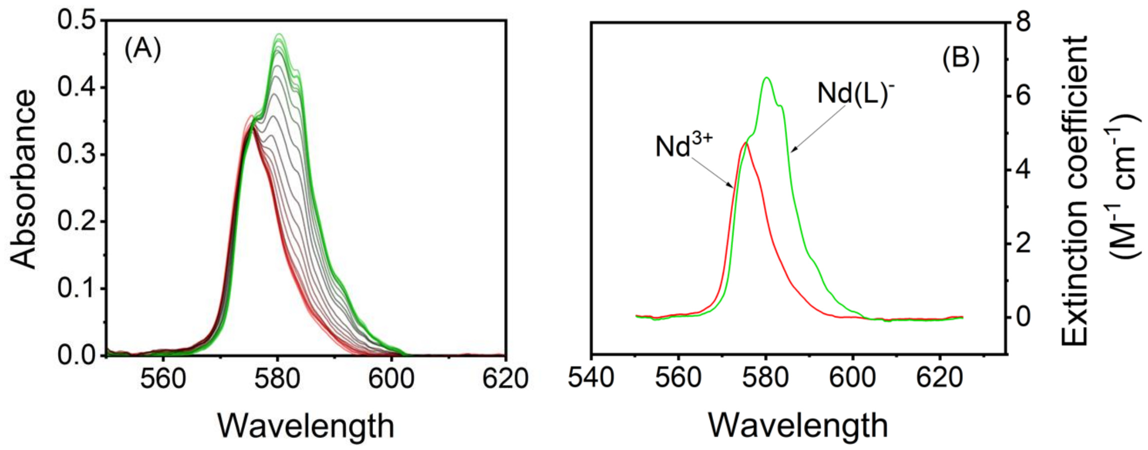

Figure S12. Spectrophotometric titration for Nd/H_4edta.

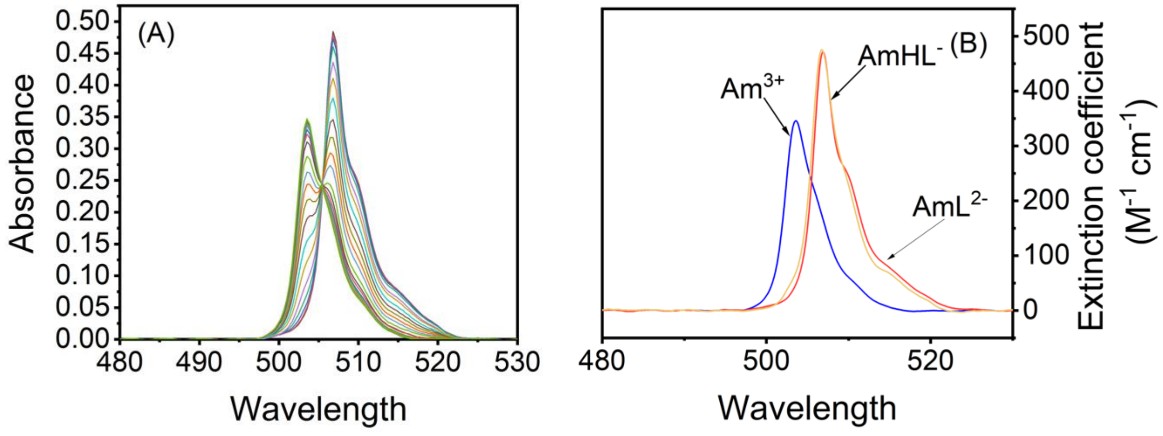

Figure S13. Spectrophotometric titration for Am/ H_4edta.

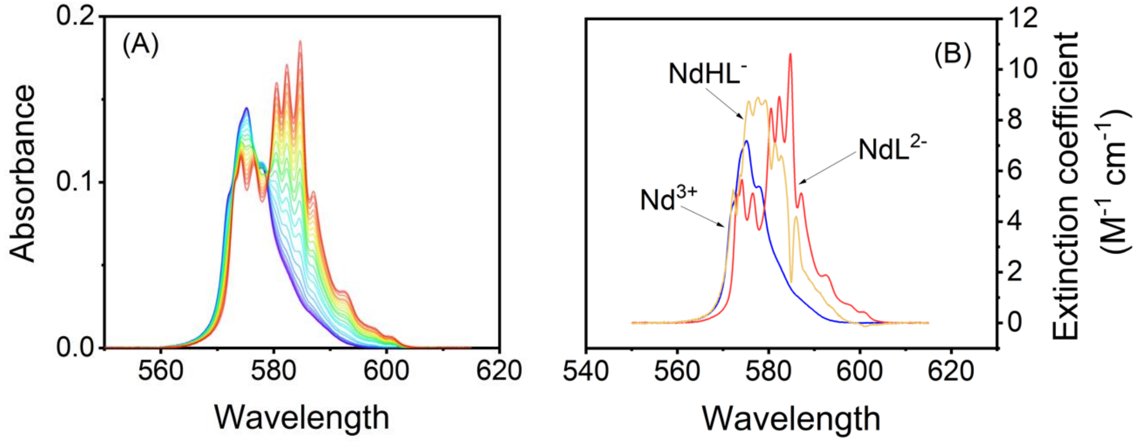

Figure S14. Spectrophotometric titration for Nd/H_5dtpa.

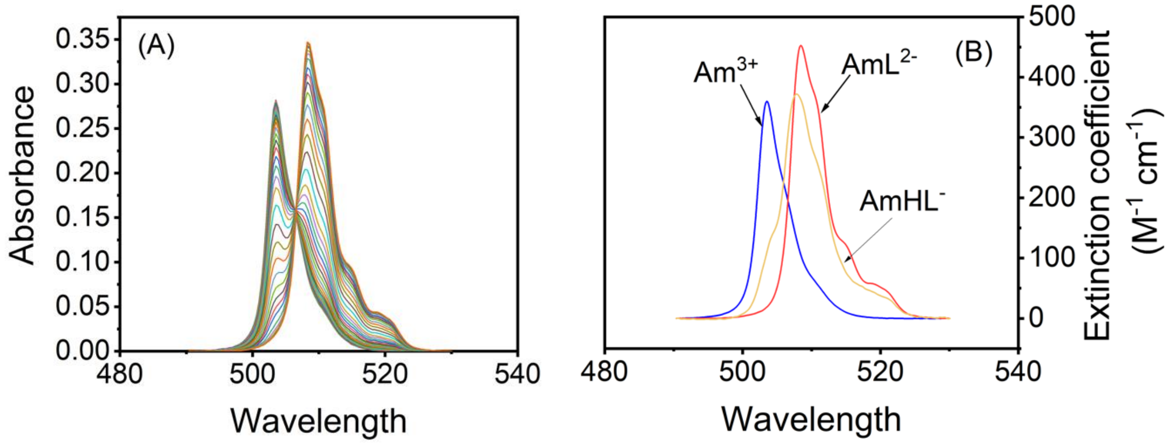

Figure S15. Spectrophotometric titration for Am/H_5dtpa.

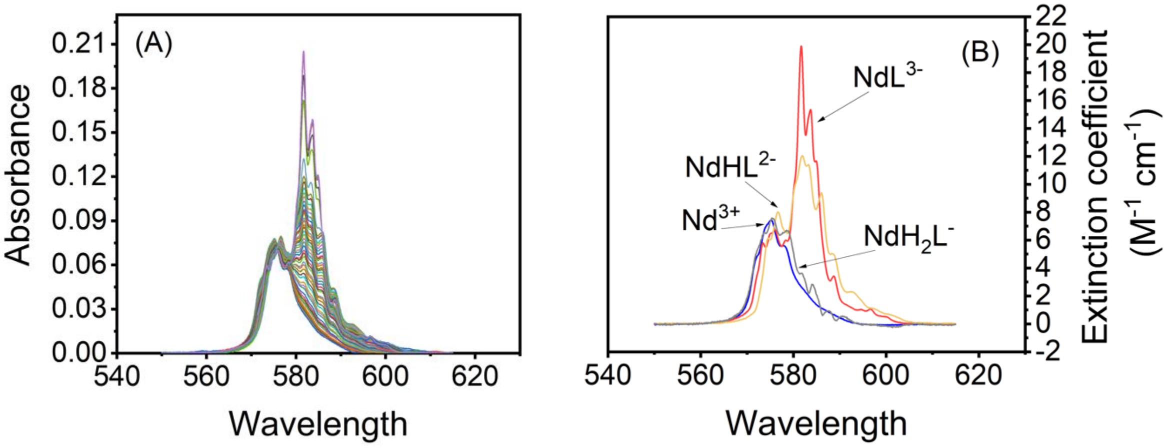

Figure S16. Spectrophotometric titration for Nd/H_6ttha.

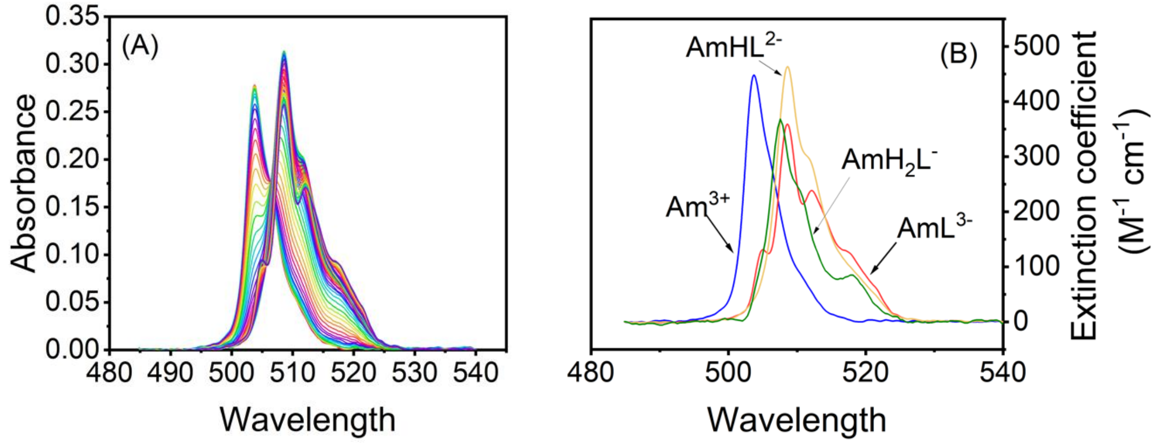

Figure S17. Spectrophotometric titration for Am/H_6ttha.

Supplemental Information References:

[S1] Abdel-Magid, A. F.; Carson, K. G.; Harris, B. D.; Maryanoff, C. A.; Shah, R. D. *J. Org. Chem.* **1996**, *61*, 3849-3862.

[S2] Jaraquemada-Pelaez, M. G.; Wang, X.; Clough, T. J.; Cao, Y.; Choundhary, N.; Emler, K.; Partick, B. O.; Orvig, C. *Dalton Trans.* **2017**, *46*, 14647-14658.

[S3] Chatterton, N.; Gateau, C.; Mazzanti, M.; Pecaut, J.; Borel, A.; Helm, L. Merbach, A. *Dalton Trans.***2005**, *6*, 1129-1135.

[S4] Becke, A. D. *J. Chem. Phys.* **1993**, *98*, 5648−5652.

[S5] Lee, C.; Yang, W.; Parr, R. G. *Phys. Rev. B: Condens. Mater. Phys.* **1988**, *37*, 785−789.

[S6] Gaussian 16, Revision A.03. Frisch, M. J.; Trucks, G. W.; Schlegel, H. B.; Scuseria, G. E.; Robb, M. A.; Cheeseman, J. R.; Scalmani, G.; Barone, V.; Men-nucci, B.; Petersson, G. A.; Nakatsuji, H.; Caricato, M.; Li, X.; Hratchian, H. P.; Izmaylov, A. F.; Bloino, J.; Zheng, G.; Sonnen-berg, J. L.; Hada, M.; Ehara, M.; Toyota, K.; Fukuda, R.; Haseg-awa, J.; Ishida, M.; Nakajima, T.; Honda, Y.; Kitao, O.; Nakai, H.; Vreven, T.; Montgomery, J. A., Jr.; Peralta, J. E.; Ogliaro, F.; Bearpark, M.; Heyd, J. J.; Brothers, E.; Kudin, K. N.; Staroverov, V. N.; Kobayashi, R.; Normand, J.; Raghavachari, K.; Rendell, A.; Burant, J. C.; Iyengar, S. S.; Tomasi, J.; Cossi, M.; Rega, N.; Millam, M. J.; Klene, M.; Knox, J. E.; Cross, J. B.; Bakken, V.; Adamo, C.; Jaramillo, J.; Gomperts, R.; Stratmann, R. E.; Yazyev, O.; Austin, A. J.; Cammi, R.; Pomelli, C.; Ochterski, J. W.; Martin, R. L.; Morokuma, K.; Zakrzewski, V. G.; Voth, G. A.; Salvador, P.; Dannenberg, J. J.; Dapprich, S.; Daniels, A. D.; Farkas, O.; Foresman, J. B.; Ortiz, J. V.; Cioslowski, J.; Fox, D. J. Gaussian, Inc., Wallingford CT, **2016**.

[S7] Nizou, G.; Favaretto, C.; Borgna, F.; Grundler, P. V.; Saffon-Merceron, N.; Platas-Iglesias, C.; Fougère, O.; Rousseaux, O.; van der Meulen, N. P.; Müller, C.; Beyler, M.; Tripier, R. *Inorg. Chem.* **2020**, *59*, 11736–11748.

[S8] Fur, M. L.; Enikő Molnár, E.; Beyler, M.; Fougère, O.; Esteban-Gómez, D.; Rousseaux, O.; Tripier, R.; Tircsó, G. ; Platas-Iglesias, C. *Inorg. Chem.* **2018**, *57*, 6932–6945.

[S9] Hu, A.; Aluicio-Sarduy, E.; Brown, V.; MacMillan, S.; Becker, K.; Barnhart, T.; Radchenko, V.; Ramogida, C.; Engle, J.; Wilson, J. *J. Am. Chem. Soc.* **2021**, *143*, 10429–10440.

[S10] Hutchison, G.; Zurek, E.; Vandermeersch, T.; Lonie, D.; Curtis, D.; Hanwell, M. *J. Cheminformatics*. **2012**, *4*, 1-17.

[S11] VandeVondele, J.; Krack, M.; Mohamed, F.; Parrinello , M.; Chassaing , T.; Hutter, J. *Comput. Phys. Commun.* **2005**, *197*, 103-128.

[S12] Goedecker, S.; Teter, M.; Hutter, J. *Phys. Rev. B.* **1996**, 54, 1703-1710.

[S13] Lippert, G.; Hutter, J.; Parrinello, M. *Mol. Phys.* **1997**, *92*, 477-487.

[S14] Perdew, J. P.; Burke, K.; Ernzerhof, M. *Phys. Rev. Lett.* **1996**, *77*, 3865-3868.

[S15] Perdew, J. P.; Burke, K.; Ernzerhof, M. *Phys. Rev. Lett.* **1997**, *78*, 1396-1396.

[S16] Grimme, S.; Antony, J.; Ehrlich, S.; Krieg, H. *J. Chem. Phys.* **2010**, *132*, 154104.
